# Racial Differences in Bleeding Risks among Patients with Atrial Fibrillation: An Ecological Epidemiological Study Comparing Korea and UK Population

**DOI:** 10.1055/a-2690-1674

**Published:** 2025-09-09

**Authors:** Dong-Seon Kang, Pil-Sung Yang, Daehoon Kim, Eunsun Jang, Hee Tae Yu, Tae-Hoon Kim, Jung Hoon Sung, Hui-Nam Pak, Gregory Y.H. Lip, Boyoung Joung

**Affiliations:** 1 Division of Cardiology, Department of Internal Medicine, Yonsei University College of Medicine, Seoul, Republic of Korea; 2 Division of Cardiology, Hallym University Sacred Heart Hospital, Hallym University College of Medicine, Anyang-si, Gyeonggi-do, Republic of Korea; 3 Division of Cardiology, CHA Bundang Medical Center, CHA University, Seongnam, Republic of Korea; 4 Liverpool Centre for Cardiovascular Science at University of Liverpool, Liverpool John Moores University and Liverpool Heart and Chest Hospital, Liverpool, United Kingdom; 5 Department of Clinical Medicine, Aalborg University, Aalborg, Denmark

**Keywords:** racial difference, atrial fibrillation, intracranial hemorrhage, gastrointestinal bleeding

## Abstract

**Background:**

Racial differences in the propensity to bleeding may be evident, with a higher risk of bleeding in Asian populations. This study aimed to assess racial differences in bleeding risk among patients with atrial fibrillation (AF) using an ecological epidemiological approach.

**Methods:**

We included patients with AF from the Korean National Health Insurance Service-Health Screening and UK Biobank who underwent health check-ups between 2006 and 2010. The analysis involved 1928 East Asians (62.1% male, median age 60.0 years) and 5917 White Europeans (71.4% male, median age 64.0 years) were analyzed. Primary outcome was composed of intracranial hemorrhage and bleeding from the gastrointestinal, respiratory, and genitourinary systems.

**Results:**

During follow-up, the primary outcome occurred in 126 East Asians and 587 White Europeans. East Asians had a 42% lower 5-year incidence rate compared with White Europeans (weighted incidence rate 1.31 vs. 2.24 per 100 person-years; incidence rate ratio 0.58, 95% confidence interval: 0.41–0.83). Contrary to the primary outcome, the incidence of intracranial hemorrhage tended to be higher among East Asians (weighted incidence rate 0.34 vs. 0.14 per 100 person-years; incidence rate ratio 2.36, 95% confidence interval: 0.88–6.37). These results persisted even in patients naïve to antithrombotic drugs. East Asians who were already taking antithrombotic drugs at baseline showed no significant difference in the incidence of the primary outcome compared with White Europeans.

**Conclusion:**

This ecological study highlights racial differences in the incidence of bleeding influenced by anatomical site and antithrombotic drug use and underscores the necessity for race-based tailored approaches.

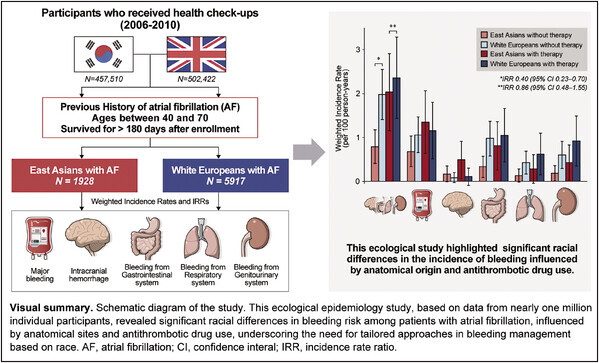

## Introduction

Atrial fibrillation (AF) is one of the most common arrhythmias requiring treatment, and stroke prevention with oral anticoagulation therapy (OAC) is one of the main pillars of AF management, as recommended in guidelines. 1 2 While OAC is used to prevent stroke and systemic embolism associated bleeding events can also increase the risk of morbidity and mortality, and in cases of serious bleeding, creates uncertainty in treatment options. 3 Various strategies have been proposed to reduce bleeding risk, including the mitigation of modifiable risk factors, reducing anticoagulant dosage, or considering alternative procedures, such as left atrial appendage occlusion. 4

However, East Asians have a different thromboembolic/bleeding profile from Western population, sometimes referred to as the East Asian Paradox, suggesting a need for medical planning and resource allocation that reflects race-specific characteristics for long-term bleeding risk management. 5 6 Yet, the 1.5 billion East Asians have been underrepresented in the pivotal cardiovascular trials, complicating the generalization of research findings; furthermore, there remains a lack of studies based on reliable statistical methods to clarify interracial differences. 7

We designed an ecological epidemiological study based on a cohort of approximately 1 million individuals extracted from South Korea and the United Kingdom to investigate the overall incidence of bleeding in patients with AF and validate the differences between the two races (East Asian vs. White European) using various analyses, and assessed bleeding risk by anatomical site. This ecological study features the characteristics of observational epidemiological studies that associate exposure and disease at the population level, rather than at the individual level. 5 8

## Methods

This retrospective study included patient-level data sourced from the Korean National Health Insurance Service-Health Screening (K-NHIS-HealS) and the UK Biobank, detailed information of which is consistent with those described in previous studies ( Supplementary Methods , available in the online version). 9 10 This study received approval from the Institutional Review Board of Yonsei University Health System (4-2022-1241) and was conducted in accordance with the Declaration of Helsinki. For the K-NHIS-HealS, identifiers were removed after cohort generation, thus waiving the need for informed consent. In the case of the UK Biobank, the use of its resources was approved by the North West Multicenter Research Ethics Committee (REC approval 21/NM/0157, application 77793), with the consent of all participants in the cohort.

### Selection of Participants

**Fig. 1:**
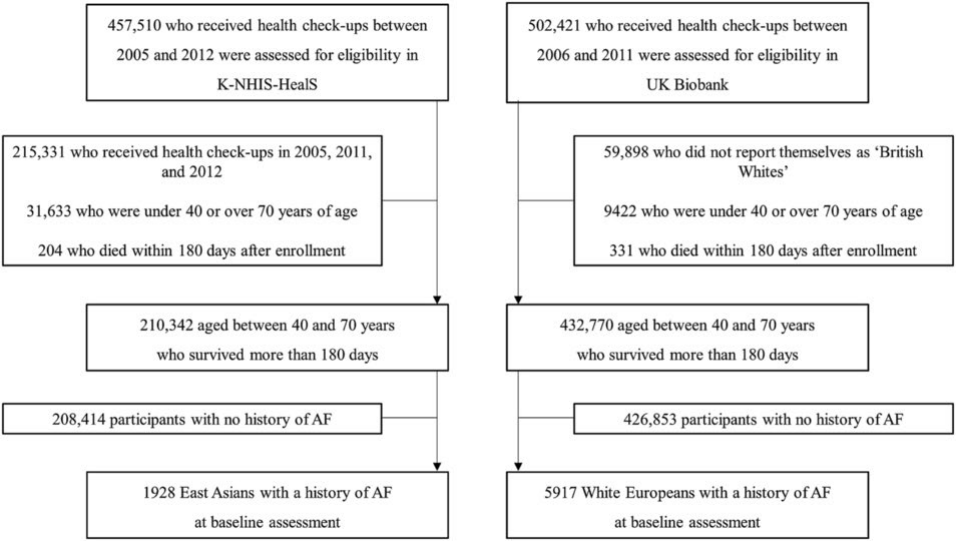
Flow chart of enrollment and analysis of the participants. This study standardized the enrollment periods for two cohorts by excluding 215,331 K-NHIS-HealS participants who underwent health check-ups in 2005, 2011, and 2012, all aged 18 years and older. Additionally, 59,898 UK Biobank participants who did not identify as “British Whites” were excluded. From both K-NHIS-HealS and the UK Biobank, participants either younger than 40 or older than 70 at enrollment (31,633 and 9,422, respectively), those who died within 180 days of enrollment (204 and 331, respectively), and those never diagnosed with AF (208,414 and 426,853, respectively) were excluded from the analysis. After applying the exclusion criteria, 1,928 East Asians and 5,917 White Europeans with AF at baseline were ultimately included in the study. AF, atrial fibrillation; K-NHIS-HealS, Korean National Health Insurance Service-Health Screening.

The detailed process was demonstrated in Fig. 1 . This study standardized the enrollment periods for two cohorts by excluding 215,331 K-NHIS-HealS participants who underwent health check-ups in 2005, 2011, and 2012, all aged 18 years and older. Additionally, 59,898 UK Biobank participants who did not identify as “British Whites” were excluded. From both K-NHIS-HealS and the UK Biobank, participants either younger than 40 or older than 70 at enrollment (31,633 and 9422, respectively), those who died within 180 days of enrollment (204 and 331, respectively), and those never diagnosed with AF (208,414 and 426,853, respectively) were excluded from the analysis.

### Covariates and Outcomes

The definitions of baseline characteristics for both cohorts are consistent with those used in previous studies ( Supplementary Table S1 , available in the online version). 5 8 Sex and race were determined based on self-reported data. Diagnostic and procedural codes from hospital visits were employed to identify underlying comorbidities, and information on medication use for cardiovascular diseases was collected from linked general practitioner electronic health records, incorporating both self-report and prescription data. 11

The primary outcome encompassed a composite of intracranial hemorrhage (ICH) and bleeding from the gastrointestinal, respiratory, and genitourinary systems. As secondary outcomes, we not only analyzed each component of the primary outcome but also considered major bleeding, defined as a composite of ICH, gastrointestinal bleeding, anemia due to bleeding, and bleeding-related death. All outcomes were defined using established ICD-10 codes from the K-NHIS-HealS and UK Biobank claims data, as well as related death records ( Supplementary Table S2 , available in the online version). 8 11 These codes had to be in the primary position; for ICH, codes for accompanying brain imaging procedures were also required. Our analysis focused on the risk of initial bleeding events, rather than recurrent bleeding events, among the patients. 12

### Statistical Analyses

The baseline characteristics of the patients were described using descriptive statistics. To evaluate differences in baseline characteristics between the two racial groups and achieve balance, overlap weighting based on propensity scores was applied. The propensity score was calculated using logistic regression that included age, sex, AF duration, systolic/diastolic blood pressure, underlying comorbidities, and concurrent medication use as covariates. The distribution of propensity scores for the two racial groups, before and after overlap weighting, was depicted in Supplementary Fig. S1 (available in the online version). A standardized mean difference of less than 0.1 was considered indicative of balance between the two racial groups for that covariate. To account for differences in follow-up duration between the two national cohorts, the 5-year incidence rates (IRs) were calculated by dividing the number of first incident events by the total person-years over five years from enrollment, and reported per 100 person-years. To compare incidence between the two racial groups, weighted IRs, incidence rate ratios (IRRs), and 95% confidence intervals (CIs) were estimated for the overlap-weighted sample, using White Europeans in the UK as the reference group.

To assess the impact of antithrombotic agents (i.e., warfarin, aspirin, P _2_ Y _12_ inhibitors) prescribed for stroke prevention on bleeding events, the additional analysis was conducted by dividing patients into those who were not taking these medications at baseline and those who were. For the former group, data were censored from the date they were prescribed antithrombotic agents during follow-up. A chi-square test was performed to determine whether the composition patterns of antithrombotic agents prescribed during follow-up differed between the two races. For the latter group, to explore the effect of antithrombotic agent types on bleeding risk, patients at baseline were further categorized into those taking only warfarin and those taking only antiplatelet agents. A Cox proportional hazards model with an interaction term for race and type of antithrombotic agent (warfarin or antiplatelet agents) was then fitted to the weighted population.

In the sensitivity analysis, to address the imbalance in baseline characteristics between the two racial groups, one-to-one propensity score matching was employed instead of overlap weighting. Second, similar to the approach for K-NHIS-HealS, only ICD-10 codes were used to define underlying comorbidities in the UK Biobank. Third, instead of using the 5-year IRs, the IRs were compared based on the number of events that occurred throughout the entire follow-up period and the total person-years for both cohorts. Lastly, subgroup analyses were conducted considering age, sex, Charlson Comorbidity Index, and obesity at baseline. When defining obesity, the body mass index (BMI) criteria suggested by Korean clinical guidelines were used for East Asians, whereas the BMI criteria suggested by World Health Organization guidelines were used for White Europeans. 13 14 All tests were two-tailed, and statistical significance was set at *p* < 0.05. All analyses were performed using R version 4.2.1 (The R Foundation, www.r-project.org ).

## Results

### Baseline Characteristics

After applying the exclusion criteria, 1928 East Asians and 5917 White Europeans with AF at baseline were ultimately included in the study. The median follow-up period (interquartile range, IQR) for East Asians was 80.6 months (61.6–88.2 months), and for White Europeans, it was 138.7 months (129.0–148.5 months). The median (IQR) age at baseline was 60.0 (54.0–65.0) years for East Asians, and 64.0 (60.0–67.0) years for White Europeans, as shown in Table 1 . The proportion of males was relatively lower in the East Asian in Korea compared with the White Europeans (62.1 vs. 71.4%, *p* < 0.001). East Asians had a higher burden of comorbidities such as hypertension, diabetes mellitus, ischemic stroke, myocardial infarction, and heart failure, and were more likely to use medications for cardiovascular diseases. Although the proportion of patients using warfarin was lower among East Asians compared with White Europeans (13.6 vs. 16.2%, *p* = 0.01), the proportion of patients using aspirin (37.1 vs. 7.0%, *p* < 0.001) and P _2_ Y _12_ inhibitors (6.2 vs. 1.3%, *p* < 0.001) were significantly higher. As presented in Supplementary Table S3 (available in the online version), after applying overlap weighting, balance was achieved between the two racial groups for all baseline characteristics, except for BMI.

**Table 1:** Baseline characteristics of study participants by race

Characteristics	East Asians	White Europeans	*p* -Value
	( *N* = 1,928)	( *N* = 5,917)	
Follow-up duration (mo)	80.6 (61.6–88.2)	138.7 (129.0–148.5)	<0.001
AF duration (mo)	32.4 (17.1–50.2)	48.3 (22.0–86.5)	<0.001
Age (y)	60.0 (54.0–65.0)	64.0 (60.0–67.0)	<0.001
Male sex	1,197 (62.1)	4,223 (71.4)	<0.001
Body mass index (kg/m ^2^ )	24.5 (22.5–26.7)	28.3 (25.5–31.8)	<0.001
<18.5	32 (1.7)	18 (0.3)	
18.5–23	543 (28.2)	505 (8.6)	
23–25	510 (26.5)	700 (11.9)	
25–30	748 (38.8)	2,483 (42.3)	
≥30	95 (4.9)	2,163 (36.9)	
Systolic blood pressure (mm Hg)	126.0 (115.0–137.0)	136.0 (124.0–149.5)	<0.001
Diastolic blood pressure (mm Hg)	80.0 (70.0–86.0)	81.5 (74.0–89.0)	<0.001
CHA _2_ DS _2_ -VASc score	2.0 (1.0–3.0)	2.0 (1.0–3.0)	<0.001
Medical history			
Hypertension	1,550 (80.4)	3,848 (65.0)	<0.001
Diabetes mellitus	364 (18.9)	786 (13.3)	<0.001
Dyslipidemia	1,218 (63.2)	2,285 (38.6)	<0.001
Ischemic stroke	374 (19.4)	358 (6.1)	<0.001
Myocardial infarction	222 (11.5)	617 (10.4)	0.19
Heart failure	580 (30.1)	890 (15.0)	<0.001
Peripheral arterial disease	174 (9.0)	113 (1.9)	<0.001
Chronic kidney disease	59 (3.1)	362 (6.1)	<0.001
End-stage kidney disease	1 (0.1)	50 (0.8)	<0.001
COPD	133 (6.9)	294 (5.0)	0.001
Malignancy	351 (18.2)	651 (11.0)	<0.001
Concurrent medication			
Antithrombotic agents	946 (49.1)	1,358 (23.0)	<0.001
Warfarin	262 (13.6)	958 (16.2)	0.01
Aspirin	716 (37.1)	414 (7.0)	<0.001
P _2_ Y _12_ inhibitors	120 (6.2)	74 (1.3)	<0.001
Statins	346 (17.9)	665 (11.2)	<0.001
ACEi/ARB	599 (31.1)	700 (11.8)	<0.001
DHP CCB	386 (20.0)	233 (3.9)	<0.001
Non-DHP CCB	158 (8.2)	115 (1.9)	<0.001
Beta blocker	509 (26.4)	598 (10.1)	<0.001
Loop diuretics	509 (26.4)	307 (5.2)	<0.001
K+ sparing diuretics	107 (5.5)	0 (0.0)	<0.001
Class IC AADs	77 (4.0)	126 (2.1)	<0.001
Class III AADs	65 (3.4)	161 (2.7)	0.16

Abbreviations: AAD, antiarrhythmic drug; ACEi, angiotensin converting enzyme inhibitor; AF, atrial fibrillation; ARB, angiotensin receptor blocker; CCB, calcium channel blocker; COPD, chronic obstructive pulmonary disease; DHP, dihydropyridine.

Notes: Data are presented as medians (interquartile range) or *N* (%). Continuous variables were analyzed using the Wilcoxon rank-sum test, and categorical variables were analyzed using the chi-square test or Fisher's exact test.

### Racial Differences in Incidence Rates of Bleeding Events

**Fig. 2:**
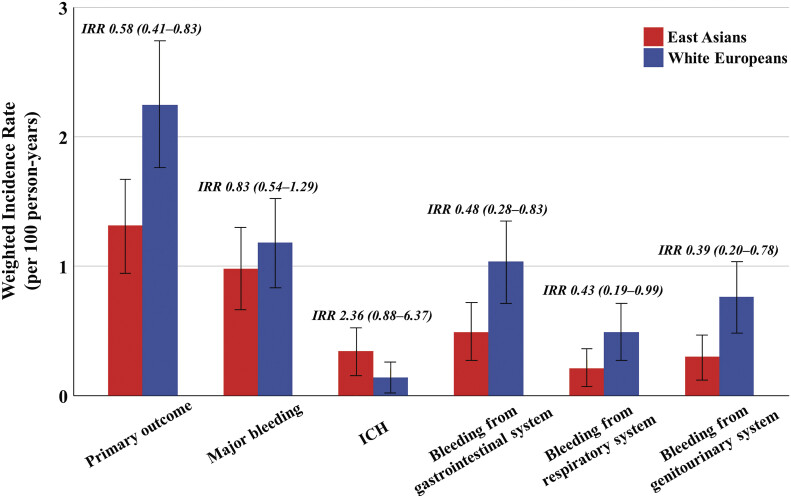
Bar graph of weighted incidence rates by race. The weighted incidence rates for the primary outcome and each component of the secondary outcome in East Asians ( *N* = 1,928) and White Europeans ( *N* = 5,917) are displayed. Error bars represent the 95% confidence intervals for each weighted incidence rate, corresponding to the values shown in Table 2 . ICH, intracranial hemorrhage; IRR, incidence rate ratio.

Over the 5 years following enrollment, 126 primary outcome events were reported among East Asians and 587 among White Europeans, respectively ( Table 2 ; Fig. 2 ). Accounting for differences in baseline characteristics, East Asians had a 42% lower incidence of the primary outcome compared with White Europeans (weighted IR 1.31 per 100 person-years vs. 2.24 per 100 person-years; IRR 0.58, 95% CI: 0.41–0.83). No significant differences in major bleeding events were observed between the two racial groups (weighted IR: 0.98 per 100 person-years vs. 1.18 per 100 person-years; IRR: 0.83, 95% CI: 0.54–1.29).

**Table 2:** Five-year incidence rates for primary and secondary outcomes by race

	East Asians	White Europeans
	( *N* = 1,928)	( *N* = 5,917)
Primary outcome		
Number of events	126	587
Person-years	8,816	27,455
Crude incidence rate (95% CI) a	1.43 (1.18–1.68)	2.14 (1.97–2.31)
Weighted incidence rate (95% CI) a	1.31 (0.94–1.67)	2.24 (1.76–2.71)
Incidence rate ratio (95% CI) b	0.58 (0.41–0.83)	1 [Reference]
Secondary outcome		
Major bleeding		
Number of events	101	291
Person-years	8,884	28,181
Crude incidence rate (95% CI) a	1.14 (0.92–1.36)	1.03 (0.91–1.15)
Weighted incidence rate (95% CI) a	0.98 (0.66–1.30)	1.18 (0.83–1.52)
Incidence rate ratio (95% CI) b	0.83 (0.54–1.29)	1 [Reference]
Intracranial hemorrhage		
Number of events	26	41
Person-years	9,027	28,753
Crude incidence rate (95% CI) a	0.29 (0.18–0.40)	0.14 (0.10–0.19)
Weighted incidence rate (95% CI) a	0.34 (0.15–0.52)	0.14 (0.02–0.26)
Incidence rate ratio (95% CI) b	2.36 (0.88–6.37)	1 [Reference]
Bleeding from gastrointestinal system		
Number of events	59	254
Person-years	8,969	28,237
Crude incidence rate (95% CI) a	0.66 (0.49–0.83)	0.90 (0.79–1.01)
Weighted incidence rate (95% CI) a	0.49 (0.27–0.72)	1.03 (0.71–1.35)
Incidence rate ratio (95% CI) b	0.48 (0.28–0.83)	1 [Reference]
Bleeding from respiratory system		
Number of events	19	129
Person-years	9,049	28,543
Crude incidence rate (95% CI) a	0.21 (0.12–0.30)	0.45 (0.37–0.53)
Weighted incidence rate (95% CI) a	0.21 (0.07–0.36)	0.49 (0.27–0.71)
Incidence rate ratio (95% CI) b	0.43 (0.19–0.99)	1 [Reference]
Bleeding from genitourinary system		
Number of events	30	211
Person-years	9,000	28,302
Crude incidence rate (95% CI) a	0.33 (0.21–0.45)	0.75 (0.64–0.85)
Weighted incidence rate (95% CI) a	0.30 (0.12–0.47)	0.76 (0.48–1.03)
Incidence rate ratio (95% CI) b	0.39 (0.20–0.78)	1 [Reference]

Abbreviation: CI, confidence interval.

a The incidence rates were calculated by dividing the number of first incident events by the total person-years over 5 years from enrollment, with 95% confidence intervals estimated using a Poisson distribution and reported per 100 person-years. Weighted incidence rates were calculated, incorporating each patient's follow-up duration and event occurrence, adjusted by their assigned weights.

b The incidence rate ratios were calculated by dividing the weighted incidence rate of East Asians by that of White Europeans. The 95% confidence intervals were derived by calculating the standard error based on the weighted number of events in each racial group.

This variation was attributable to contrasting race-specific tendencies in bleeding. The incidence of ICH was over 2-fold higher in East Asians compared with White Europeans (weighted IR: 0.34 per 100 person-years vs. 0.14 per 100 person-years; IRR: 2.36, 95% CI: 0.88–6.37), whereas the incidence of bleeding from the gastrointestinal system was 52% lower in East Asians (weighted IR: 0.49 per 100 person-years vs. 1.03 per 100 person-years; IRR: 0.48, 95% CI: 0.28–0.83). Bleeding from the respiratory system (weighted IR: 0.21 per 100 person-years vs. 0.49 per 100 person-years; IRR: 0.43, 95% CI: 0.19–0.99) and the genitourinary system (weighted IR: 0.30 per 100 person-years vs. 0.76 per 100 person-years; IRR: 0.39, 95% CI: 0.20–0.78) also occurred 57 and 61% less frequently in East Asians, contributing to the results in the primary outcome.

The anatomical dependence of bleeding tendencies was also reflected in their relative proportions ( Supplementary Fig. S2 , available in the online version). The total number of all reported bleeding events was 134 in East Asians and 635 in White Europeans. While both cohorts predominantly experienced bleeding from the gastrointestinal system and genitourinary system, followed by the third most common event being ICH in East Asians (19.4 vs. 6.5%), and bleeding from the respiratory system in White Europeans (14.2 vs. 20.3%).

### The Impact of Antithrombotic Agents on Bleeding Events

**Fig. 3:**
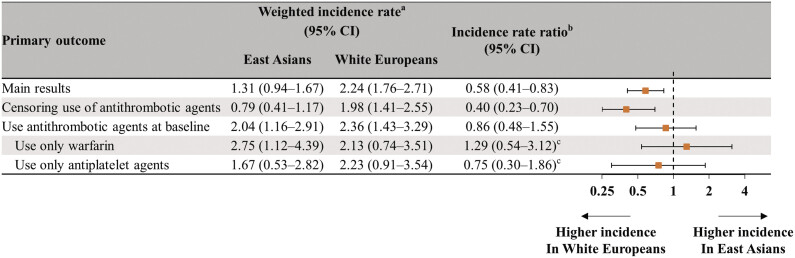
Results on the primary outcome by race according to the use of antithrombotic agents. To analyze the results censoring for the use of antithrombotic agents, 982 East Asians and 4,559 White Europeans not using antithrombotic agents at baseline were included. To analyze the results while on antithrombotic agents, 946 East Asians and 1,358 White Europeans who were already using antithrombotic agents at baseline were included. Additionally, separate analyses were performed for those using only warfarin (186 East Asians, 903 White Europeans) and those using only antiplatelet agents (684 East Asians, 400 White Europeans) at baseline. Error bars indicate 95% confidence intervals for the incidence rate ratios. ^a^ Weighted incidence rates were calculated by dividing the number of first incident events by the total person-years over 5 years from enrollment, with adjustments for assigned weights. The 95% confidence intervals were estimated using a Poisson distribution and reported per 100 person-years. ^b^ The incidence rate ratios were calculated by dividing the weighted incidence rate of East Asians by that of White Europeans. The 95% CIs were derived by calculating the standard error based on the weighted number of events in each racial group. ^c^ The results of the Cox proportional hazards model, including the interaction term for race and type of antithrombotic agent in the weighted population, showed no significant interaction ( *p* for interaction = 0.26). CI, confidence interval.

At baseline, 982 East Asians and 4,559 White Europeans were not using antithrombotic agents. During follow-up, 346 of these East Asians received a total of 452 antithrombotic prescriptions (99 for warfarin, 203 for aspirin, and 150 for P _2_ Y _12_ inhibitors), and 859 of these White Europeans received a total of 1,130 prescriptions (648 for warfarin, 316 for aspirin, and 166 for P _2_ Y _12_ inhibitors). Differences in the composition patterns of prescribed antithrombotic agents between the two races were also seen ( *p* < 0.001). Even when censoring for the use of antithrombotic agents during follow-up, East Asians still exhibited a lower overall IR for the primary outcome compared with White Europeans (weighted IR: 0.79 per 100 person-years vs. 1.98 per 100 person-years; IRR: 0.40, 95% CI: 0.23–0.70; Fig. 3 ). While events of ICH were still relatively more frequent (weighted IR: 0.17 per 100 person-years vs. 0.08 per 100 person-years; IRR: 2.08, 95% CI: 0.37–11.60), whereas events of bleeding from the gastrointestinal system were less frequent (weighted IR: 0.33 per 100 person-years vs. 0.98 per 100 person-years; IRR: 0.34, 95% CI: 0.15–0.79), consistent with the main findings across all outcomes ( Supplementary Fig. S3 , available in the online version).

For patients who were already prescribed antithrombotic agents at baseline, the relatively lower incidence of the primary outcome in East Asians was attenuated (weighted IR: 2.04 per 100 person-years vs. 2.36 per 100 person-years; IRR: 0.86, 95% CI: 0.48–1.55), and the higher incidence of ICH became more prominent (weighted IR: 0.49 per 100 person-years vs. 0.11 per 100 person-years; IRR: 4.54, 95% CI: 0.61–33.62). Detailed results are presented in Fig. 3 and Supplementary Fig. S3 (available in the online version). An exploratory analysis confined to OAC users only and antiplatelet users only at baseline, did not show significant statistical interactions between the two antithrombotic strategies, except for bleeding from the respiratory system, which had a very small number of events ( *p* for interaction < 0.001).

### Sensitivity and Subgroup Analyses

After successfully adjusting for differences in baseline characteristics between the two cohorts through one-to-one propensity score matching and conducting the same analysis ( Supplementary Tables S4, S5 , available in the online version), using only ICD-10 codes to define comorbidities in the UK Biobank ( Supplementary Table S6 , available in the online version), calculating IRs based on the total number of events and person-years over the entire follow-up period ( Supplementary Table S7 , available in the online version), and analyzing subgroups ( Supplementary Figs. S4–S9 , available in the online version), the results were consistent with the main findings.

## Discussion

The principal findings of this ecological epidemiological study, using patient-level data from South Korea and the United Kingdom, are as follows: First, East Asians with AF had a relatively lower incidence of overall bleeding, as represented by the primary outcome, compared with White Europeans. Second, similar to the general population, ICH was more frequent in East Asians than in White Europeans. Third, current users of antithrombotic agents for stroke prevention due to AF were associated with an attenuation of the relatively lower bleeding risk observed in East Asians, whereas the higher incidence of ICH became more prominent.

### Racial Differences in Bleeding Tendencies and Patterns

Our study demonstrates that East Asians have a lower overall bleeding incidence compared with White Europeans, with notable variations based on the anatomical origin of the bleeding. Past studies have focused on the burden of cardiovascular comorbidities and the pharmacokinetic and pharmacodynamic characteristics of antithrombotic agents to explain the different bleeding tendencies among races. 6 Importantly, approaches targeting current users to find clues about racial differences are not free from the bias of selecting “survivors” with little or no bleeding tendencies. Additionally, the results of this study, which censored antithrombotic prescription initiation to provide a pure perspective on bleeding tendencies in untreated populations, support the need for further exploration of racial characteristics per se.

A lack of consistent methodologies and individual-level data has hindered comparisons between races. 15 Our series of studies indicates that classifying bleeding events by anatomical origin may provide clues to explaining this phenomenon. 8 Similar to the general population, the incidence of ICH was higher in East Asians, whereas the incidence of bleeding from the gastrointestinal system was lower in East Asians. The susceptibility of East Asians to ICH is a well-known phenomenon from pivotal trials, although the specific mechanisms remain unclear. 16 17 Several factors are thought to be related. Intracranial atherosclerosis is about twice as prevalent in East Asians (30–50 vs. 15–30%), 18 and the prevalence of microbleeds, which can increase the risk of ICH by about 3-fold, is also significantly higher in Asian patients compared with non-Asian patients (68.4 vs. 56.9%). 19 Additionally, the incidence of cerebral arteriovenous malformations has been reported to be higher in Asians compared with Western populations, 20 all of which may contribute to the increased likelihood of ICH in East Asians. Additionally, genetic polymorphisms in the *ACE* , *RAGE* , and *CD36* genes—implicated in vascular constriction, vascular remodeling, and endothelial dysfunction—have been reported to contribute to the development of hypertension and atherosclerosis, and are associated with an increased risk of ICH. 21 However, these genetic variants alone are insufficient to fully explain the risk of ICH in East Asians.

Regarding bleeding from the gastrointestinal system, the incidence of *Helicobacter pylori* infection is gradually decreasing in East Asians due to regular endoscopic examinations, which is common because of the high prevalence of gastric cancer. 22 In contrast, diverticulosis is still more commonly reported in Western populations, partially contributing to the differences in bleeding incidence between races. 23 From a public health perspective, effectively preventing bleeding events with limited medical resources requires considering race-specific epidemiological information on bleeding, including the anatomical origins of bleeding. In Korea, the government provides biennial upper endoscopy as a reimbursable service under the national health insurance for individuals aged over 40 years, serving as an example of a public health policy aimed at risk factor management. 24 This approach can ultimately lead to improved long-term outcomes. 1 25

### Influence of Antithrombotic Agents on Racial Differences in Bleeding Risk

First, it should be noted that White Europeans were more commonly treated with warfarin (16.2%) as an antithrombotic strategy, whereas East Asians primarily used antiplatelet agents such as aspirin (37.1%) and P _2_ Y _12_ inhibitors (6.2%). A previous individual-patient data meta-analysis reported that when compared with aspirin, warfarin reduced the risk of stroke by 38%, while doubling the risk of major bleeding. 26 The fact that warfarin did not reduce vascular- or all-cause death led physicians to prescribe warfarin to patients with relatively high stroke risk and to use aspirin for those with lower risk. 26 Although a randomized clinical trial in elderly AF patients showed the noninferiority of warfarin compared with aspirin for bleeding risks, 27 several limitations—such as focusing only on Europeans, a low number of bleeding events, frequent crossover between treatment strategies, and a high proportion of patients already on warfarin at enrollment—combined with the reasons described below, were insufficient to alleviate the concerns of physicians managing the specific AF population subgroup referred to as East Asians.

Despite a tendency to maintaining lower international normalized ratio (INR) values, it is well-known that East Asians have a higher incidence of bleeding, particularly ICH, compared with White Europeans, and the sequelae and mortality of bleeding also tended to be greater. 28 Although the exact reasons for this phenomenon are not clearly understood, enhanced pharmacokinetics of warfarin in East Asians is often considered. The lower frequency of variant alleles such as *CYP2C9* * *2* and *CYP2C9* * *3* , which are known to reduce the catalytic activity of cytochrome P450 2C9, in Japanese results in a reduced maintenance dose and increased bleeding. 29 Additionally, the haplotype frequency of the gene affecting hepatic vitamin K epoxide reductase, which influences the synthesis of various coagulation factors, was 89% in East Asians compared with only 42% in Caucasians, partly explaining the narrower therapeutic index of warfarin in East Asians. 30 Beyond genetic predisposition, it is also argued that the lower lean body mass, faster rate of renal function decline, and the relatively frequent intake of herbal materials in East Asian countries affects the blood levels of warfarin, making it difficult to maintain an appropriate time in therapeutic range. 31 32 Reflecting these concerns, Japanese guidelines currently recommend an optimal INR of 1.6 to 2.6 for elderly AF patients over the age of 70. 33 Similarly, for nonvitamin K antagonist oral anticoagulants (NOACs), the differences in thrombotic and bleeding profiles observed between races in this study may generate the hypothesis of defining race-specific standard doses—illustrated by the example of Japan, where pharmacokinetic modeling data supported adopting a 15 mg standard dose of rivaroxaban instead of 20 mg. 1

Antiplatelet agents are not effective alternatives for East Asians. The BAFTA and ACTIVE W studies revealed that antiplatelet agents do not reduce bleeding events compared with warfarin and were inferior for stroke prevention. 27 Moreover, for some P _2_ Y _12_ inhibitors, the dose required to reach the same blood concentration was lower in East Asians than in Caucasians, leading to more frequent ICH and bleeding from gastrointestinal system in East Asians, supporting the findings of this study. 18 The most preferred NOACs in contemporary guidelines have demonstrated greater reductions in the risks of major bleeding, clinically relevant nonmajor bleeding, and gastrointestinal bleeding in Asians compared with warfarin. 34 However, the fact remains that the absolute ICH incidence in Asians taking NOACs is still higher than in Caucasians, highlighting the necessity for race-specific bleeding prevention strategies that should not be overlooked in the advancement of treatment options. 6 28

## Limitations

Our study has several limitations. First, due to the nature of an ecological observational study, residual confounding factors may still exist. Because this study was based on claim-based data, it was not possible to obtain detailed clinical information such as laboratory test results (e.g., time in therapeutic range, hemoglobin levels during bleeding events), specific imaging findings, the success of hemostatic procedures or surgeries, or the presence of shock during bleeding events. As a result, there are inherent limitations in assessing bleeding severity and in establishing causality based on the observed results. Second, we relied on ICD-10 codes to define comorbidities, which introduces the possibility of coding errors. Additionally, the use of self-reported information to define comorbidities and medication use may result in further measurement errors. Third, unlike the K-NHIS-HealS data, which is considered representative of the general population, the UK Biobank data consists of voluntary participants who may not represent the general population. 35 Fourth, participants younger than 40 years or older than 70 years were excluded to ensure appropriate matching between the two racial groups and to minimize the influence of extreme values. Therefore, our findings cannot be generalized to these excluded age groups. Fifth, there was no information provided on the changes in anthropometric and comorbidity variables over time. Sixth, the East Asians in this study all resided in Korea, limiting the applicability of our conclusions to Asians in other regions.

## Conclusion

This ecological epidemiological study of patients with AF also showed that East Asians tend to have fewer overall bleeding events compared with Caucasians. However, East Asians still exhibited a greater susceptibility to ICH, and the lower overall bleeding incidence in East Asians was attenuated in prevalent users of antithrombotic agents. This suggests that the anatomical origin of bleeding and the use of antithrombotic agents are significant factors in explaining racial differences in bleeding tendencies.

## Notes

**What is known about this topic?**

- Patients with atrial fibrillation (AF) take antithrombotic agents to prevent stroke, but this inevitably involves bleeding events. According to previous pivotal trials related to AF, East Asians appear to have a distinct thromboembolic/bleeding profile compared with Western populations.
- The evaluation of bleeding events has mainly been limited to intracranial hemorrhage among various anatomical origins. Furthermore, East Asians are often an underrepresented population in studies, making it difficult to generalize study conclusions and find reliable, unified statistical methods to identify racial differences.

**What does this paper add?**

- This ecological study was based on individual-level data extracted from the Korean National Health Insurance Service-Health Screening and UK Biobank.
- Based on unified definitions and statistical methods between international cohorts, East Asians have a 42% lower overall incidence of bleeding compared with White Europeans. However, similar to the findings of previous pivotal trials, East Asians remain more vulnerable to intracranial hemorrhage. When antithrombotic agents were already being used, the relatively lower overall bleeding tendency observed in East Asians was attenuated.
- Our findings support that the bleeding profile of East Asians with AF differs from that of Western populations, and emphasize the need for race-specific bleeding prevention strategies.
