# Supplementary material for: Racial Differences in Bleeding Risks among Patients with Atrial Fibrillation: An Ecological Epidemiological Study Comparing Korea and UK Population
Source: Thromb Haemost. 2025 Sep 9;126(7):754–64. doi: 10.1055/a-2690-1674 (PMC13286097; doi:10.1055/a-2690-1674)
Supplement: Supplementary file 1 — Supplementary Material [file 10-1055-a-2690-1674_26978380.pdf]

**SUPPLEMENTARY**

# Racial differences in bleeding risks among patients with atrial fibrillation: An ecological epidemiological study comparing Korea and UK population

Dong-Seon Kang, MD, Pil-Sung Yang, MD, Daehoon Kim, MD, Eunsun Jang, MS, Hee Tae Yu, MD, PhD, Tae-Hoon Kim, MD, Jung Hoon Sung, MD, Hui-Nam Pak, MD, PhD, Gregory Y.H. Lip, MD, PhD, Boyoung Joung, MD, PhD

| Contents | Page |
|---|---|
| **Supplemental Methods** | 2 |
| **Table S1.** Definitions used for defining the comorbidities. | 3–4 |
| **Table S2.** Definitions used for defining outcomes. | 5 |
| **Table S3.** Baseline characteristics of study participants by race after overlap weighting. | 6 |
| **Table S4.** Baseline characteristics of study participants by race after one-to-one propensity score matching. | 7 |
| **Table S5.** Five-year incidence rates for primary and secondary outcomes by race after one-to-one propensity score matching. | 8 |
| **Table S6.** Five-year incidence rates for primary and secondary outcomes by race when comorbidities were defined only by ICD-10 codes. | 9 |
| **Table S7.** Incidence rates for primary and secondary outcomes by race when the number of events and person-years were investigated over the entire follow-up period. | 10 |
| **Figure S1.** Distributions of the propensity scores before and after overlap weighting. | 11 |
| **Figure S2.** Proportional distribution of components within the primary outcome. | 12 |
| **Figure S3.** Results on the secondary outcomes by race according to the use of antithrombotic agents. | 13 |
| **Figure S4.** Subgroup analyses for the primary outcome. | 14 |
| **Figure S5.** Subgroup analyses for major bleeding. | 15 |
| **Figure S6.** Subgroup analyses for intracranial hemorrhage. | 16 |
| **Figure S7.** Subgroup analyses for bleeding from gastrointestinal system. | 17 |
| **Figure S8.** Subgroup analyses for bleeding from respiratory system. | 18 |
| **Figure S9.** Subgroup analyses for bleeding from genitourinary system. | 19 |



Thrombosis and Haemostasis | DOI 10.1055/a-2690-1674 | © The Author(s).

## Supplemental Methods

***Data Source***

The Korea National Health Insurance Service-Health Screening (K-NHIS-HealS) data, distributed since 2015, is based on individuals mandatorily enrolled in the National Health Insurance Service (NHIS), covering 97.1% of the total Korean population. The K-NHIS-HealS data comprises a random sample of 10% of health screening participants aged 40-80, totaling 558,147 Koreans. We selected those who received health screenings and qualified for K-NHIS-HealS between 2005 and 2012. Participants visited designated centers every two years from 2005 onwards, providing basic medical information such as sociodemographic and anthropometric data, comorbidities, family history, radiological and blood tests. They were followed until death, immigration, or the end of the study (December 31, 2013), whichever came first. Information on death (date and primary cause) was verified using death certificates from the National Population Registry of the Korea National Statistical Office.

The UK Biobank is a prospective population-based cohort study involving 502,422 participants aged 37-73, recruited from England, Scotland, and Wales between March 2006 and August 2010. Participants were invited to designated centers to provide basic medical information similar to the K-NHIS-HealS survey. They were followed until the point of UK Biobank disqualification (death or immigration) or the end of the study (March 31, 2021, for England and Scotland, and February 28, 2018, for Wales), whichever came first. Information on death was verified using death certificates from the National Health Service Information Centre/National Health Service Central Register Scotland.



Thrombosis and Haemostasis | DOI 10.1055/a-2690-1674 | © The Author(s).

**Table S1.** Definitions used for defining the comorbidities.

| Comorbidities | K-NHIS-HealS | | UK Biobank | |
|---|---|---|---|---|
| | Definitions | Used codes or conditions | Definitions | Used codes or conditions |
| Atrial fibrillation | Defined from diagnosis[a] | ICD-10: I48 | Defined from UK Biobank self-report or diagnosis[a] | Self-reported non-cancer illness code: 1471, 1483<br>ICD-10: I48 |
| Hypertension | Defined from diagnosis[a] plus treatment | ICD-10: I10, I11, I12, I13, I15<br>Treatment: all kinds of blood pressure lowering medications (>1 month). | Defined from UK Biobank self-report or diagnosis[a] | Self-reported non-cancer illness code: 1065, 1072<br>ICD-10: I10, I11, I12, I13, I15 |
| Diabetes mellitus | Defined from diagnosis[a] plus treatment | ICD-10: E10, E11, E12, E13, E14<br>Treatment: all kinds of oral antidiabetics and insulin | Defined from UK Biobank self-report or diagnosis[a] | Self-reported non-cancer illness code: 1220, 1222, 1223, 1521<br>ICD-10: E10, E11, E12, E13, E14 |
| Ischemic stroke | Defined from diagnosis[a] | ICD-10: I63, I64 | Defined from UK Biobank self-report or diagnosis[a] | Self-reported non-cancer illness code: 1583<br>ICD-10: I63, I64 |
| Transient ischemic attack | Defined from diagnosis[a] | ICD-10: G45 | Defined from UK Biobank self-report or diagnosis[a] | Self-reported non-cancer illness code: 1082<br>ICD-10: G45 |
| Myocardial infarction (MI) | Defined from diagnosis[a] | ICD-10: I21, I22, I25.2 | Defined from UK Biobank self-report or diagnosis[a] | Self-reported non-cancer illness code: 1075<br>ICD-10: I21, I22, I25.2 |
| Heart failure | Defined from diagnosis[a] | ICD-10: I11.0, I50, I97.1 | Defined from UK Biobank self-report or diagnosis[a] | Self-reported non-cancer illness code: 1076<br>ICD-10: I11.0, I50, I97.1 |
| Peripheral arterial disease | Defined from diagnosis[a] | ICD-10: I70, I71 | Defined from UK Biobank self-report or diagnosis[a] | Self-reported non-cancer illness code: 1067, 1087<br>ICD-10: I70, I71 |
| Chronic kidney disease | Defined from eGFR (if laboratory value was not available, diagnosis code was used) | eGFR <60 mL/min per 1.73 $m^2$<br>ICD-10: N18, N19 | Defined from eGFR (if laboratory value was not available, self-report or diagnosis code was used) | eGFR <60 mL/min per 1.73 $m^2$<br>Self-reported non-cancer illness code:1192, 1194<br>ICD-10: N18, N19 |



Thrombosis and Haemostasis | DOI 10.1055/a-2690-1674 | © The Author(s).

| | | | | |
|---|---|---|---|---|
| End stage renal disease (ESRD) | Defined from national registry for severe illness or history of renal replacement therapy (hemodialysis, peritoneal dialysis, or kidney transplant) | Patients with ESRD undergoing chronic dialysis or received a kidney transplant. | Defined from UK Biobank self-report or procedure codes related to renal replacement therapy (hemodialysis, peritoneal dialysis, or kidney transplant) | Self-reported non-cancer illness code: 1193, 1195, 1580, 1581, 1582<br>Procedure codes: L74, M01, M02.3, M08.4, M17, X40, X41, X42 |
| Chronic obstructive pulmonary disease | Defined from diagnosis[a] plus treatment | ICD-10: J42, J43(except J43.0), J44<br>Treatment: SABA, SAMA, LABA, LAMA, ICS, ICS+LABA, or methylxanthine (>1 month). | Defined from UK Biobank self-report or diagnosis[a] | Self-reported non-cancer illness code: 1112, 1113, 1472<br>ICD-10: J42, J43(except J43.0), J44 |
| Malignancy | Defined from diagnoses of cancer (non-benign) | ICD-10: C00-C97 | Defined from UK Biobank self-report or diagnoses of cancer (non-benign) | Self-reported cancer illness code: all<br>ICD-10: C00-C97 |

[a]To ensure accuracy, comorbidities were established based on more than one hospital-inpatient or two outpatients (= primary care in United Kingdom) records of ICD-10 codes in the database.
ESRD, end-stage renal disease; eGFR, estimated glomerular filtration rate; ICD, International Classification of Disease; ICS, inhaled corticosteroid; K-NHIS-HealS, Korean National Health Insurance Service-Health Screening; LABA, long-acting beta2 agonist; LAMA, long-acting muscarinic antagonist; MI, myocardial infarction; SABA, short-acting beta2 agonist; SAMA, short-acting muscarinic agonist.



Thrombosis and Haemostasis | DOI 10.1055/a-2690-1674 | © The Author(s).

**Table S2.** Definitions used for defining outcomes.

| | K-NHIS-HealS | | UK Biobank | |
|---|---|---|---|---|
| | **Definitions** | **Used codes or conditions** | **Definitions** | **Used codes or conditions** |
| Major bleeding | ICH, bleeding from gastrointestinal system, or anemia caused by bleeding, or related death | ICD-10: I60-I62, K25-28 (subcodes 0-2 and 4-6 only), K92.0, K92.1, K92.2, K62.5, I85.0, I98.3, D62 | ICH, bleeding from gastrointestinal system, or anemia caused by bleeding, or related death | ICD-10: I60-I62, K25-28 (subcodes 0-2 and 4-6 only), K92.0, K92.1, K92.2, K62.5, I85.0, I98.3, D62 |
| ICH | Defined from hospital-inpatient diagnosis of ICH with concomitant brain imaging studies, or related death | ICD-10: I60-I62<br>Procedure codes for brain imaging: HE101, HE201, HE501, HF101, HF201, HF102, HF202, HE135, HE235, HE535, HA451, HA461, HA471 | Defined from hospital-inpatient diagnosis of ICH with concomitant brain imaging studies, or related death | ICD-10: I60-I62<br>Procedure codes for brain imaging: U051, U052, U053, U114 |
| Bleeding from gastrointestinal system | Defined from hospital-inpatient diagnosis, or related death | ICD-10: K25-28 (subcodes 0-2 and 4-6 only), K92.0, K92.1, K92.2, K62.5, I85.0, I98.3 | Defined from hospital-inpatient diagnosis, or related death | ICD-10: K25-28 (subcodes 0-2 and 4-6 only), K92.0, K92.1, K92.2, K62.5, I85.0, I98.3 |
| Bleeding from respiratory system | Defined from hospital-inpatient diagnosis, or related death | ICD-10: R04, J942 | Defined from hospital-inpatient diagnosis, or related death | ICD-10: R04, J942 |
| Bleeding from genitourinary system | Defined from hospital-inpatient diagnosis, or related death | ICD-10: N02, R31 | Defined from hospital-inpatient diagnosis, or related death | ICD-10: N02, R31 |

ICD, International Classification of Disease; ICH, intracranial hemorrhage; K-NHIS-HealS, Korean National Health Insurance Service-Health Screening.



Thrombosis and Haemostasis | DOI 10.1055/a-2690-1674 | © The Author(s).

**Table S3.** Baseline characteristics of study participants by race after overlap weighting.

| Characteristics | East Asians (N=807) | Whites Europeans (N=807) | P value | SMD |
|---|---|---|---|---|
| Follow-up duration (months) | 79.0 (61.1–88.1) | 138.3 (129.3–148.4) | <0.001 | 2.32 |
| AF duration (months) | 35.0 (18.9–53.8) | 28.1 (13.4–53.0) | <0.001 | <0.001 |
| Age (years) | 61.0 (55.0–66.0) | 62.0 (56.0–65.0) | 0.57 | <0.001 |
| Male sex | 522.4 (64.7) | 522.4 (64.7) | 1.00 | <0.001 |
| Body mass index ($kg/m^2$) | 24.3 (22.3–26.3) | 28.9 (25.9–32.5) | <0.001 | 1.18 |
| <18.5 | 16.1 (2.0) | 3.1 (0.4) | | |
| 18.5–23 | 244.0 (30.2) | 61.6 (7.7) | | |
| 23–25 | 222.9 (27.6) | 86.9 (10.9) | | |
| 25–30 | 292.5 (36.2) | 316.4 (39.6) | | |
| ≥30 | 31.4 (3.9) | 331.7 (41.5) | | |
| Systolic blood pressure (mmHg) | 130.0 (118.0–139.0) | 128.0 (117.0–139.5) | 0.45 | <0.001 |
| Diastolic blood pressure (mmHg) | 80.0 (70.0–88.0) | 79.0 (72.0–86.5) | 0.94 | <0.001 |
| $CHA_2DS_2$-VASc score[a] | 2.0 (1.0–3.0) | 2.0 (1.0–3.0) | 0.06 | 0.05 |
| Medical history | | | | |
| Hypertension | 565.7 (70.1) | 565.7 (70.1) | 1.00 | <0.001 |
| Diabetes mellitus | 121.4 (15.0) | 121.4 (15.0) | 1.00 | <0.001 |
| Dyslipidemia | 411.3 (51.0) | 411.3 (51.0) | 1.00 | <0.001 |
| Ischemic stroke | 93.9 (11.6) | 93.9 (11.6) | 1.00 | <0.001 |
| Myocardial infarction | 84.4 (10.5) | 84.4 (10.5) | 1.00 | <0.001 |
| Heart failure | 170.7 (21.2) | 170.7 (21.2) | 1.00 | <0.001 |
| Peripheral arterial disease | 32.2 (4.0) | 32.2 (4.0) | 1.00 | <0.001 |
| Chronic kidney disease | 28.3 (3.5) | 28.3 (3.5) | 1.00 | <0.001 |
| End-stage kidney disease | 0.7 (0.1) | 0.7 (0.1) | 1.00 | <0.001 |
| COPD | 51.4 (6.4) | 51.4 (6.4) | 1.00 | <0.001 |
| Malignancy | 121.4 (15.0) | 121.4 (15.0) | 1.00 | <0.001 |
| Concurrent medication | | | | |
| Antithrombotic agents | 248.0 (30.7) | 247.8 (30.7) | 0.99 | 0.001 |
| Warfarin | 114.3 (14.2) | 114.3 (14.2) | 1.00 | <0.001 |
| Aspirin | 142.0 (17.6) | 142.0 (17.6) | 1.00 | <0.001 |
| $P_2Y_{12}$ inhibitors | 24.1 (3.0) | 24.1 (3.0) | 1.00 | <0.001 |
| Statins | 127.6 (15.8) | 127.6 (15.8) | 1.00 | <0.001 |
| ACEi/ARB | 161.1 (20.0) | 161.1 (20.0) | 1.00 | <0.001 |
| DHP CCB | 81.4 (10.1) | 81.4 (10.1) | 1.00 | <0.001 |
| Non-DHP CCB | 37.9 (4.7) | 37.9 (4.7) | 1.00 | <0.001 |
| Beta blocker | 134.9 (16.7) | 134.9 (16.7) | 1.00 | <0.001 |
| Loop diuretics | 93.5 (11.6) | 93.5 (11.6) | 1.00 | <0.001 |
| K+ sparing diuretics | 0.0 (0.0) | 0.0 (0.0) | 0.32 | <0.001 |
| Class IC AADs | 21.9 (2.7) | 18.0 (2.2) | 0.34 | 0.03 |
| Class III AADs | 18.9 (2.3) | 26.3 (3.3) | 0.11 | 0.06 |

Data are presented as medians (interquartile range) or No. (%). Continuous variables were analyzed using the Wilcoxon rank-sum test, and categorical variables were analyzed using the Chi-square test or Fisher's exact test.
AAD, antiarrhythmic drug; ACEi, angiotensin converting enzyme inhibitor; AF, atrial fibrillation; ARB, angiotensin receptor blocker; CCB, calcium channel blocker; COPD, chronic obstructive pulmonary disease; DHP, dihydropyridine; SMD, standardized mean difference.



Thrombosis and Haemostasis | DOI 10.1055/a-2690-1674 | © The Author(s).

**Table S4.** Baseline characteristics of study participants by race after one-to-one propensity score matching.

| Characteristics | East Asians (N=1073) | White Europeans (N=1073) | P value | SMD |
|---|---|---|---|---|
| Follow-up duration (months) | 79.3 (61.3–88.2) | 138.2 (128.1–148.7) | <0.001 | 2.25 |
| AF duration (months) | 34.1 (18.7–53.2) | 27.9 (12.8–53.4) | 0.001 | 0.02 |
| Age (years) | 60.0 (55.0–65.0) | 62.0 (56.0–65.0) | 0.22 | 0.03 |
| Male sex | 694 (64.7) | 700 (65.2) | 0.82 | 0.01 |
| Body mass index (kg/m$^2$) | 24.4 (22.3–26.4) | 29.3 (26.0–32.9) | <0.001 | 1.22 |
| <18.5 | 20 (1.9) | 4 (0.4) | | |
| 18.5–23 | 328 (30.6) | 76 (7.1) | | |
| 23–25 | 277 (25.8) | 106 (10.0) | | |
| 25–30 | 405 (37.7) | 406 (38.2) | | |
| ≥30 | 43 (4.0) | 472 (44.4) | | |
| Systolic blood pressure (mmHg) | 130.0 (118.0–138.0) | 126.5 (117.0–137.5) | 0.96 | 0.01 |
| Diastolic blood pressure (mmHg) | 80.0 (70.0–87.0) | 79.0 (72.5–86.5) | 0.76 | 0.01 |
| $CHA_2DS_2$-VASc score | 2.0 (1.0–3.0) | 2.0 (1.0–3.0) | 0.01 | 0.09 |
| Medical history | | | | |
| Hypertension | 759 (70.7) | 768 (71.6) | 0.70 | 0.02 |
| Diabetes mellitus | 174 (16.2) | 177 (16.5) | 0.91 | 0.01 |
| Dyslipidemia | 566 (52.7) | 566 (52.7) | 1.00 | <0.001 |
| Ischemic stroke | 123 (11.5) | 132 (12.3) | 0.59 | 0.03 |
| Myocardial infarction | 112 (10.4) | 116 (10.8) | 0.83 | 0.01 |
| Heart failure | 223 (20.8) | 229 (21.3) | 0.79 | 0.01 |
| Peripheral arterial disease | 52 (4.8) | 46 (4.3) | 0.61 | 0.03 |
| Chronic kidney disease | 35 (3.3) | 37 (3.4) | 0.91 | 0.01 |
| End-stage kidney disease | 1 (0.1) | 0 (0.0) | 1.00 | 0.04 |
| COPD | 64 (6.0) | 66 (6.2) | 0.93 | 0.01 |
| Malignancy | 178 (16.6) | 160 (14.9) | 0.31 | 0.05 |
| Concurrent medication | | | | |
| Antithrombotic agents | 325 (30.3) | 330 (30.8) | 0.85 | 0.01 |
| Warfarin | 150 (14.0) | 145 (13.5) | 0.80 | 0.01 |
| Aspirin | 185 (17.2) | 199 (18.5) | 0.46 | 0.03 |
| $P_2Y_{12}$ inhibitors | 30 (2.8) | 34 (3.2) | 0.70 | 0.02 |
| Statins | 176 (16.4) | 168 (15.7) | 0.68 | 0.02 |
| ACEi/ARB | 219 (20.4) | 225 (21.0) | 0.79 | 0.01 |
| DHP CCB | 116 (10.8) | 107 (10.0) | 0.57 | 0.03 |
| Non-DHP CCB | 48 (4.5) | 47 (4.4) | 1.00 | 0.01 |
| Beta blocker | 186 (17.3) | 182 (17.0) | 0.86 | 0.01 |
| Loop diuretics | 126 (11.7) | 134 (12.5) | 0.64 | 0.02 |
| K+ sparing diuretics | 0.0 (0.0) | 0.0 (0.0) | - | <0.001 |
| Class IC AADs | 26 (2.4) | 24 (2.2) | 0.89 | 0.01 |
| Class III AADs | 21 (2.0) | 37 (3.4) | 0.05 | 0.09 |

Data are presented as medians (interquartile range) or No. (%). Continuous variables were analyzed using the Wilcoxon rank-sum test, and categorical variables were analyzed using the Chi-square test or Fisher's exact test.
AAD, antiarrhythmic drug; ACEi, angiotensin converting enzyme inhibitor; AF, atrial fibrillation; ARB, angiotensin receptor blocker; CCB, calcium channel blocker; COPD, chronic obstructive pulmonary disease; DHP, dihydropyridine; SMD, standardized mean difference.



Thrombosis and Haemostasis | DOI 10.1055/a-2690-1674 | © The Author(s).

**Table S5.** Five-year incidence rates for primary and secondary outcomes by race after one-to-one propensity score matching.

| | East Asians (N=1073) | White Europeans (N=1073) |
|---|---|---|
| **Primary outcome** | | |
| Number of events | 68 | 117 |
| Person-years | 4926 | 4949 |
| Incidence rate (95% CI)[a] | 1.38 (1.05–1.71) | 2.36 (1.94–2.79) |
| Incidence rate ratio (95% CI)[b] | 0.58 (0.43–0.79) | 1 [Reference] |
| **Secondary outcome** | | |
| **Major bleeding** | | |
| Number of events | 55 | 64 |
| Person-years | 4953 | 5080 |
| Incidence rate (95% CI)[a] | 1.11 (0.82–1.40) | 1.26 (0.95–1.57) |
| Incidence rate ratio (95% CI)[b] | 0.88 (0.61–1.26) | 1 [Reference] |
| **Intracranial hemorrhage** | | |
| Number of events | 17 | 6 |
| Person-years | 5028 | 5,214 |
| Incidence rate (95% CI)[a] | 0.34 (0.18–0.50) | 0.12 (0.02–0.21) |
| Incidence rate ratio (95% CI)[b] | 2.94 (1.16–7.45) | 1 [Reference] |
| **Bleeding from gastrointestinal bleeding** | | |
| Number of events | 30 | 58 |
| Person-years | 5007 | 5089 |
| Incidence rate (95% CI)[a] | 0.60 (0.38–0.81) | 1.14 (0.85–1.43) |
| Incidence rate ratio (95% CI)[b] | 0.53 (0.34–0.82) | 1 [Reference] |
| **Bleeding from respiratory system** | | |
| Number of events | 10 | 29 |
| Person-years | 5046 | 5158 |
| Incidence rate (95% CI)[a] | 0.20 (0.08–0.32) | 0.56 (0.36–0.77) |
| Incidence rate ratio (95% CI)[b] | 0.35 (0.17–0.72) | 1 [Reference] |
| **Bleeding from genitourinary system** | | |
| Number of events | 16 | 42 |
| Person-years | 5028 | 5124 |
| Incidence rate (95% CI)[a] | 0.32 (0.16–0.47) | 0.82 (0.57–1.07) |
| Incidence rate ratio (95% CI)[b] | 0.39 (0.22–0.69) | 1 [Reference] |

[a]The incidence rates were calculated by dividing the number of first incident events by the total person-years over five years from enrollment, with 95% confidence intervals estimated using a Poisson distribution and reported per 100 person-years.
[b]The incidence rate ratios were calculated by dividing the incidence rate of East Asians by that of White Europeans. The 95% confidence intervals were derived by calculating the standard error based on the number of events in each racial group
CI, confidence interval.



Thrombosis and Haemostasis | DOI 10.1055/a-2690-1674 | © The Author(s).

**Table S6.** Five-year incidence rates for primary and secondary outcomes by race when comorbidities were defined only by ICD-10 codes.

| | East Asians (N=1928) | White Europeans (N=5917) |
|---|---|---|
| **Primary outcome** | | |
| Number of events | 126 | 587 |
| Person-years | 8816 | 27455 |
| Crude incidence rate (95% CI)[a] | 1.43 (1.18–1.68) | 2.14 (1.97–2.31) |
| Weighted incidence rate (95% CI)[a] | 1.28 (0.90–1.67) | 2.33 (1.81–2.85) |
| Incidence rate ratio (95% CI)[b] | 0.55 (0.38–0.80) | 1 [Reference] |
| **Secondary outcome** | | |
| **Major bleeding** | | |
| Number of events | 101 | 291 |
| Person-years | 8884 | 28,181 |
| Crude incidence rate (95% CI)[a] | 1.14 (0.92–1.36) | 1.03 (0.91–1.15) |
| Weighted incidence rate (95% CI)[a] | 0.97 (0.64–1.31) | 1.20 (0.83–1.56) |
| Incidence rate ratio (95% CI)[b] | 0.81 (0.51–1.29) | 1 [Reference] |
| **Intracranial hemorrhage** | | |
| Number of events | 26 | 41 |
| Person-years | 9027 | 28,753 |
| Crude incidence rate (95% CI)[a] | 0.29 (0.18–0.40) | 0.14 (0.10–0.19) |
| Weighted incidence rate (95% CI)[a] | 0.33 (0.13–0.52) | 0.15 (0.02–0.28) |
| Incidence rate ratio (95% CI)[b] | 2.16 (0.77–6.09) | 1 [Reference] |
| **Bleeding from gastrointestinal bleeding** | | |
| Number of events | 59 | 254 |
| Person-years | 8969 | 28,237 |
| Crude incidence rate (95% CI)[a] | 0.66 (0.49–0.83) | 0.90 (0.79–1.01) |
| Weighted incidence rate (95% CI)[a] | 0.51 (0.27–0.75) | 1.05 (0.70–1.39) |
| Incidence rate ratio (95% CI)[b] | 0.49 (0.28–0.87) | 1 [Reference] |
| **Bleeding from respiratory system** | | |
| Number of events | 19 | 129 |
| Person-years | 9049 | 28,543 |
| Crude incidence rate (95% CI)[a] | 0.21 (0.12–0.30) | 0.45 (0.37–0.53) |
| Weighted incidence rate (95% CI)[a] | 0.20 (0.05–0.36) | 0.55 (0.30–0.79) |
| Incidence rate ratio (95% CI)[b] | 0.38 (0.16–0.90) | 1 [Reference] |
| **Bleeding from genitourinary system** | | |
| Number of events | 30 | 211 |
| Person-years | 9000 | 28,302 |
| Crude incidence rate (95% CI)[a] | 0.33 (0.21–0.45) | 0.75 (0.64–0.85) |
| Weighted incidence rate (95% CI)[a] | 0.28 (0.11–0.46) | 0.76 (0.47–1.05) |
| Incidence rate ratio (95% CI)[b] | 0.37 (0.18–0.78) | 1 [Reference] |

[a]The incidence rates were calculated by dividing the number of first incident events by the total person-years over five years from enrollment, with 95% confidence intervals estimated using a Poisson distribution and reported per 100 person-years. Weighted incidence rates were calculated, incorporating each patient's follow-up duration and event occurrence, adjusted by their assigned weights.
[b]The incidence rate ratios were calculated by dividing the weighted incidence rate of East Asians by that of White Europeans. The 95% confidence intervals were derived by calculating the standard error based on the weighted number of events in each racial group.
CI, confidence interval; ICD, International Classification of Disease.



Thrombosis and Haemostasis | DOI 10.1055/a-2690-1674 | © The Author(s).

Thieme

**Table S7.** Incidence rates for primary and secondary outcomes by race when the number of events and person-years were investigated over the entire follow-up period.

| | East Asians (N=1928) | White Europeans (N=5917) |
|---|---|---|
| **Primary outcome** | | |
| Number of events | 159 | 1231 |
| Person-years | 11,363 | 58,174 |
| Crude incidence rate (95% CI)[a] | 1.40 (1.18–1.62) | 2.12 (2.00–2.23) |
| Weighted incidence rate (95% CI)[a] | 1.27 (0.95–1.60) | 2.08 (1.76–2.40) |
| Incidence rate ratio (95% CI)[b] | 0.61 (0.46–0.82) | 1 [Reference] |
| **Secondary outcome** | | |
| **Major bleeding** | | |
| Number of events | 126 | 667 |
| Person-years | 11,486 | 61,516 |
| Crude incidence rate (95% CI)[a] | 1.10 (0.91–1.29) | 1.08 (1.00–1.17) |
| Weighted incidence rate (95% CI)[a] | 0.94 (0.66–1.21) | 1.11 (0.89–1.34) |
| Incidence rate ratio (95% CI)[b] | 0.84 (0.59–1.20) | 1 [Reference] |
| **Intracranial hemorrhage** | | |
| Number of events | 33 | 98 |
| Person-years | 11,742 | 64,301 |
| Crude incidence rate (95% CI)[a] | 0.28 (0.19–0.38) | 0.15 (0.12–0.18) |
| Weighted incidence rate (95% CI)[a] | 0.30 (0.15–0.46) | 0.13 (0.05–0.20) |
| Incidence rate ratio (95% CI)[b] | 2.35 (1.08–5.09) | 1 [Reference] |
| **Bleeding from gastrointestinal bleeding** | | |
| Number of events | 74 | 580 |
| Person-years | 11,627 | 61,813 |
| Crude incidence rate (95% CI)[a] | 0.64 (0.49–0.78) | 0.94 (0.86–1.01) |
| Weighted incidence rate (95% CI)[a] | 0.49 (0.29–0.68) | 0.99 (0.77–1.20) |
| Incidence rate ratio (95% CI)[b] | 0.49 (0.31–0.78) | 1 [Reference] |
| **Bleeding from respiratory system** | | |
| Number of events | 32 | 319 |
| Person-years | 11,774 | 63,238 |
| Crude incidence rate (95% CI)[a] | 0.27 (0.18–0.37) | 0.50 (0.45–0.56) |
| Weighted incidence rate (95% CI)[a] | 0.23 (0.10–0.37) | 0.52 (0.36–0.67) |
| Incidence rate ratio (95% CI)[b] | 0.45 (0.23–0.86) | 1 [Reference] |
| **Bleeding from genitourinary system** | | |
| Number of events | 39 | 441 |
| Person-years | 11,690 | 62,125 |
| Crude incidence rate (95% CI)[a] | 0.33 (0.23–0.44) | 0.71 (0.64–0.78) |
| Weighted incidence rate (95% CI)[a] | 0.34 (0.17–0.50) | 0.67 (0.50–0.85) |
| Incidence rate ratio (95% CI)[b] | 0.50 (0.29–0.87) | 1 [Reference] |

[a]The incidence rates were calculated by dividing the number of first incident events by the total person-years over five years from enrollment, with 95% confidence intervals estimated using a Poisson distribution and reported per 100 person-years. Weighted incidence rates were calculated, incorporating each patient's follow-up duration and event occurrence, adjusted by their assigned weights.
[b]The incidence rate ratios were calculated by dividing the weighted incidence rate of East Asians by that of White Europeans. The 95% confidence intervals were derived by calculating the standard error based on the weighted number of events in each racial group.
CI, confidence interval.



Thrombosis and Haemostasis | DOI 10.1055/a-2690-1674 | © The Author(s).

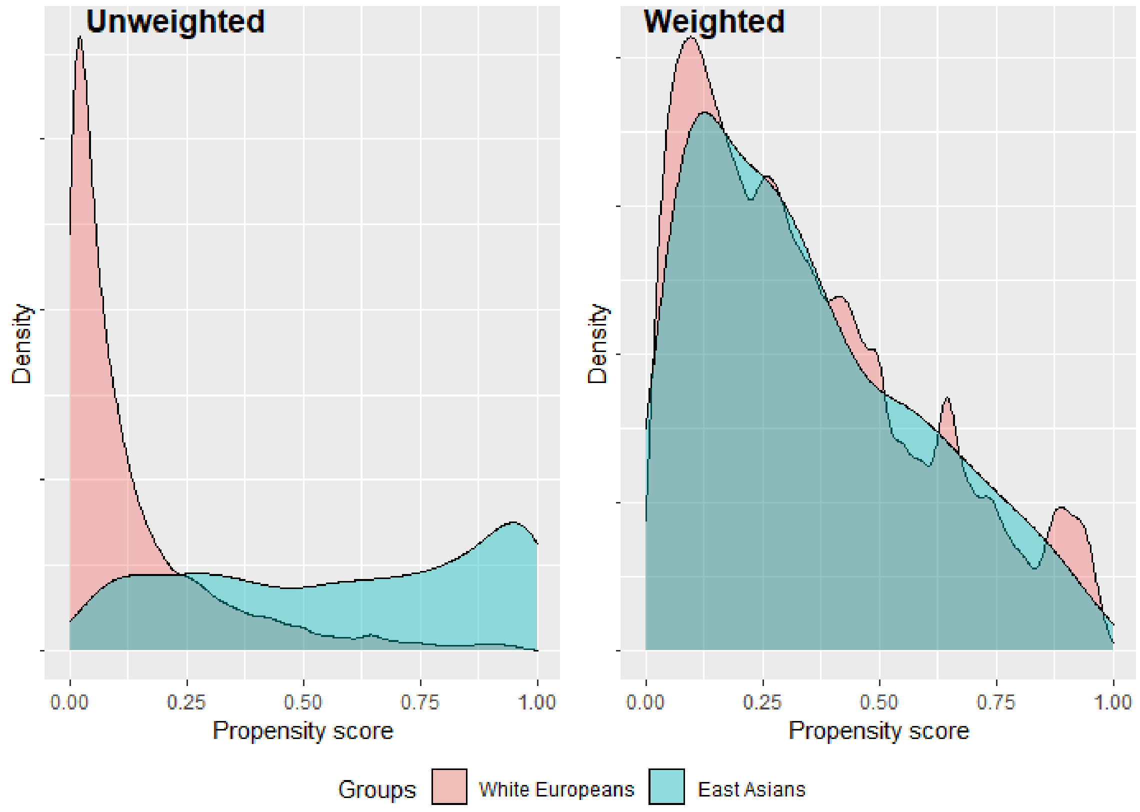


**Figure S1.** Distributions of the propensity scores before and after overlap weighting.

To evaluate differences in baseline characteristics between the two racial groups and achieve balance, overlap weighting based on propensity scores was applied. The propensity score was calculated using logistic regression that included age, sex, AF duration, systolic/diastolic blood pressure, underlying comorbidities, and concurrent medication use as covariates. This figure illustrates the distribution of propensity scores within the two racial groups..
AF, atrial fibrillation.



Thrombosis and Haemostasis | DOI 10.1055/a-2690-1674 | © The Author(s).

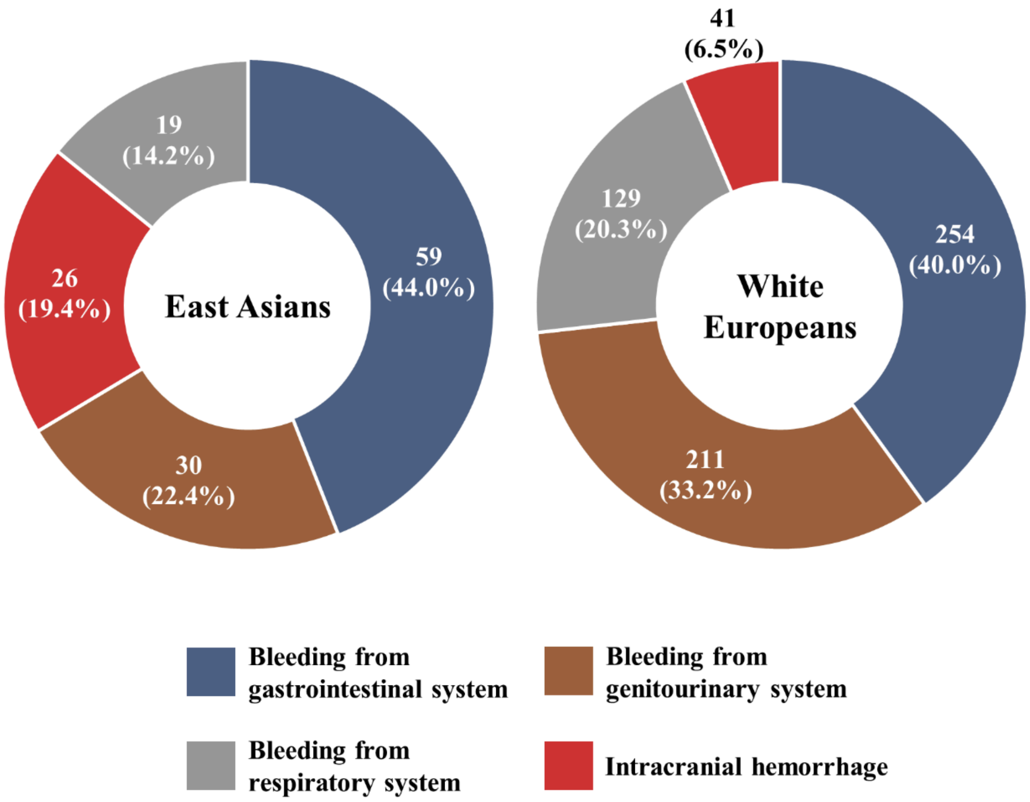


**Figure S2.** Proportional distribution of components within the primary outcome.

The total number of all reported bleeding events was 134 in East Asians and 635 in White Europeans. This figure illustrates the proportion, in No. (%), of each anatomical origin of bleeding within the total bleeding events.



Thrombosis and Haemostasis | DOI 10.1055/a-2690-1674 | © The Author(s).

| Major bleeding | Weighted incidence rate[a] (95% CI) East Asians | Weighted incidence rate[a] (95% CI) White Europeans | Incidence rate ratio[b] (95% CI) |
|---|---|---|---|
| Main results | 0.98 (0.66–1.30) | 1.18 (0.83–1.52) | 0.83 (0.54–1.29) |
| Censoring use of antithrombotic agents | 0.68 (0.33–1.03) | 1.06 (0.65–1.47) | 0.64 (0.34–1.23) |
| Use antithrombotic agents at baseline | 1.35 (0.64–2.06) | 1.16 (0.51–1.80) | 1.16 (0.54–2.50) |
| Use only warfarin | 1.55 (0.34–2.77) | 0.90 (0.01–1.79) | 1.73 (0.49–6.09)[c] |
| Use only antiplatelet agents | 1.31 (0.30–2.33) | 1.23 (0.27–2.20) | 1.07 (0.35–3.20)[c] |
| **Intracranial hemorrhage** | | | |
| Main results | 0.34 (0.15–0.52) | 0.14 (0.02–0.26) | 2.36 (0.88–6.37) |
| Censoring use of antithrombotic agents | 0.17 (-0.01–0.35) | 0.08 (-0.03–0.20) | 2.08 (0.37–11.60) |
| Use antithrombotic agents at baseline | 0.49 (0.06–0.91) | 0.11 (-0.09–0.30) | 4.54 (0.61–33.62) |
| Use only warfarin | 0.90 (-0.01–1.82) | 0.14 (-0.21–0.48) | 6.54 (0.44–97.70)[c] |
| Use only antiplatelet agents | 0.31 (-0.18–0.80) | 0.06 (-0.15–0.26) | 5.69 (0.11–301.59)[c] |
| **Bleeding from gastrointestinal system** | | | |
| Main results | 0.49 (0.27–0.72) | 1.03 (0.71–1.35) | 0.48 (0.28–0.83) |
| Censoring use of antithrombotic agents | 0.33 (0.09–0.57) | 0.98 (0.58–1.37) | 0.34 (0.15–0.79) |
| Use antithrombotic agents at baseline | 0.81 (0.26–1.36) | 1.05 (0.44–1.66) | 0.77 (0.31–1.88) |
| Use only warfarin | 0.74 (-0.09–1.57) | 0.77 (-0.05–1.59) | 0.96 (0.20–4.55)[c] |
| Use only antiplatelet agents | 0.88 (0.06–1.71) | 1.18 (0.23–2.12) | 0.75 (0.22–2.58)[c] |
| **Bleeding from respiratory system** | | | |
| Main results | 0.21 (0.07–0.36) | 0.49 (0.27–0.71) | 0.43 (0.19–0.99) |
| Censoring use of antithrombotic agents | 0.13 (-0.02–0.28) | 0.43 (0.17–0.69) | 0.30 (0.08–1.13) |
| Use antithrombotic agents at baseline | 0.28 (-0.04–0.61) | 0.62 (0.15–1.10) | 0.46 (0.12–1.78) |
| Use only warfarin | 0.61 (-0.14–1.36) | 0.42 (-0.19–1.02) | 1.46 (0.22–9.70)[d] |
| Use only antiplatelet agents | 0.01 (-0.05–0.06) | 0.43 (-0.14–1.00) | 0.01 (0.001–7468)[d] |
| **Bleeding from genitourinary system** | | | |
| Main results | 0.30 (0.12–0.47) | 0.76 (0.48–1.03) | 0.39 (0.20–0.78) |
| Censoring use of antithrombotic agents | 0.18 (0.01–0.36) | 0.60 (0.29–0.91) | 0.30 (0.10–0.93) |
| Use antithrombotic agents at baseline | 0.43 (0.03–0.83) | 0.92 (0.35–1.49) | 0.47 (0.16–1.43) |
| Use only warfarin | 0.50 (-0.18–1.19) | 1.18 (0.16–2.21) | 0.42 (0.08–2.14)[c] |
| Use only antiplatelet agents | 0.44 (-0.14–1.02) | 0.79 (0.02–1.56) | 0.56 (0.11–2.88)[c] |

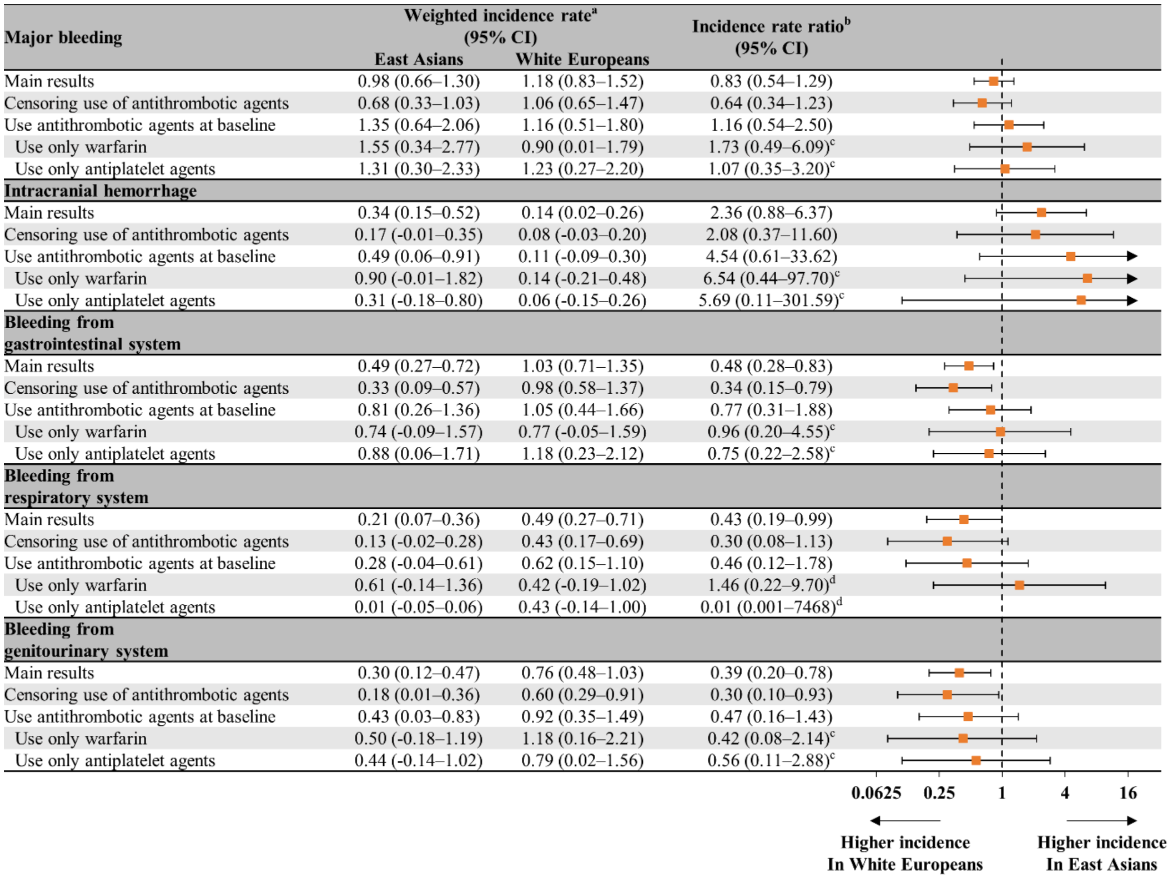


**Figure S3.** Results on the secondary outcomes by race according to the use of antithrombotic agents.

To analyze the results censoring for the use of antithrombotic agents, 982 East Asians and 4559 White Europeans not using antithrombotic agents at baseline were included. To analyze the results while on antithrombotic agents, 946 East Asians and 1358 White Europeans who were already using antithrombotic agents at baseline were included. Additionally, separate analyses were performed for those using only warfarin (186 East Asians, 903 White Europeans) and those using only antiplatelet agents (684 East Asians, 400 White Europeans) at baseline. Error bars indicate 95% confidence intervals for the incidence rate ratios.

[a]Weighted incidence rates were calculated by dividing the number of first incident events by the total person-years over five years from enrollment, with adjustments for assigned weights. The 95% confidence intervals were estimated using a Poisson distribution and reported per 100 person-years.

[b]The incidence rate ratios were calculated by dividing the weighted incidence rate of East Asians by that of White Europeans. The 95% CIs were derived by calculating the standard error based on the weighted number of events in each racial group.

[c]The results of the Cox proportional hazards model, including the interaction term for race and type of antithrombotic agent in the weighted population, showed no significant interaction (P for interaction = 0.26).

[d]The results of the Cox proportional hazards model, including the interaction term for race and type of antithrombotic agent in the weighted population, showed a significant (P for interaction < 0.001).

CI, confidence interval.



Thrombosis and Haemostasis | DOI 10.1055/a-2690-1674 | © The Author(s).

| Primary outcome | East Asians | White Europeans | IRR (95% CI) |
|---|---|---|---|
| | Weighted IR[a] (95% CI) | Weighted IR[a] (95% CI) | |
| **Age** | | | |
| < 65 | 1.09 (0.69–1.49) | 2.05 (1.50–2.60) | 0.53 (0.34–0.84) |
| ≥ 65 | 1.83 (1.02–2.63) | 2.84 (1.83–3.84) | 0.64 (0.37–1.13) |
| **Sex** | | | |
| Men | 1.40 (0.92–1.87) | 2.27 (1.67–2.88) | 0.61 (0.40–0.95) |
| Women | 1.14 (0.56–1.73) | 2.11 (1.32–2.90) | 0.54 (0.29–1.02) |
| **Charlson comorbidity index** | | | |
| < 3 | 0.94 (0.55–1.33) | 1.79 (1.26–2.33) | 0.52 (0.31–0.87) |
| ≥ 3 | 2.03 (1.07–2.99) | 3.46 (2.21–4.71) | 0.58 (0.32–1.07) |
| **Obesity** | | | |
| No | 1.07 (0.63–1.51) | 2.16 (1.54–2.78) | 0.49 (0.30–0.82) |
| Yes[b] | 1.62 (0.97–2.27) | 2.41 (1.62–3.20) | 0.67 (0.40–1.13) |

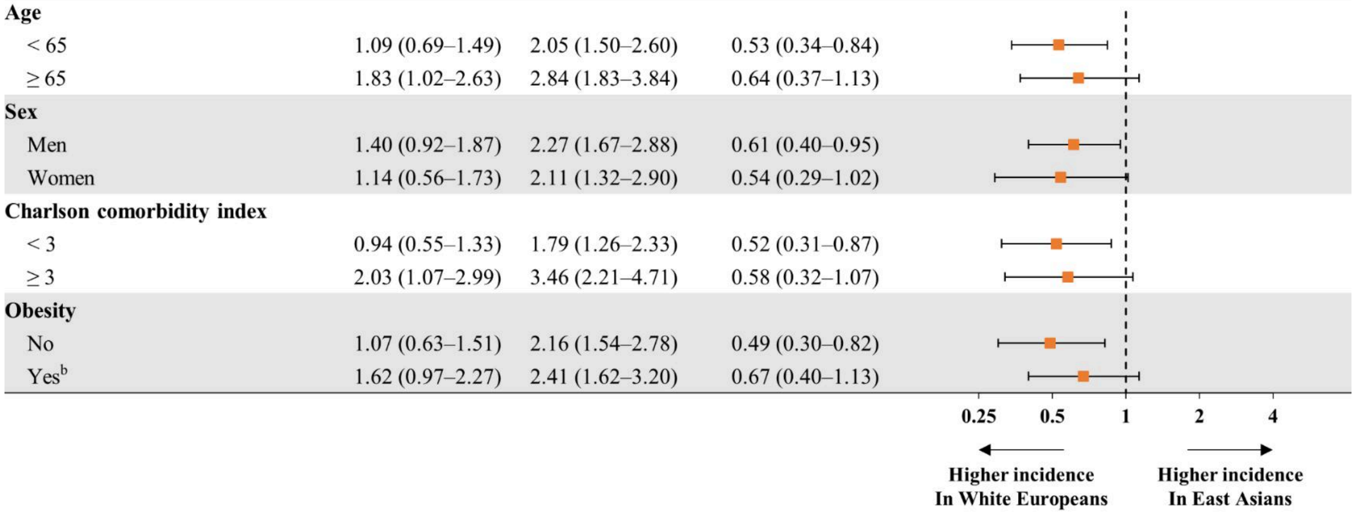


**Figure S4.** Subgroup analyses for the primary outcome.

This figure presents the results of subgroup analyses, dividing East Asians and White Europeans into various categories: those under 65 years of age (1411 and 3265, respectively) and those 65 and older (517 and 2652, respectively); men (1197 and 4223, respectively) and women (731 and 1694, respectively); those with a Charlson comorbidity index below 3 (918 and 4903, respectively) and those with an index of 3 or above (662 and 1158, respectively); and participants classified as non-obese (1085 and 3706, respectively) or obese (843 and 2163, respectively). Participants with missing values for the variables under analysis were excluded from the corresponding analysis. Error bars indicate 95% confidence intervals for the incidence rate ratio.

[a]Weighted incidence rates were calculated by dividing the number of first incident events by the total person-years over five years from enrollment, with adjustments for assigned weights. The 95% confidence intervals were estimated using a Poisson distribution and reported per 100 person-years.

[b]Obesity was defined as a body mass index of 25 kg/m² or higher for East Asians, and 30 kg/m² or higher for White Europeans.

CI, condfidence interval; IR, incidence rate; IRR, incidence rate ratio.



Thrombosis and Haemostasis | DOI 10.1055/a-2690-1674 | © The Author(s).

| Major bleeding | East Asians Weighted IR[a] (95% CI) | White Europeans Weighted IR[a] (95% CI) | IRR (95% CI) |
|---|---|---|---|
| **Age** | | | |
| < 65 | 0.80 (0.46–1.15) | 1.07 (0.68–1.46) | 0.75 (0.43–1.32) |
| ≥ 65 | 1.43 (0.72–2.14) | 1.56 (0.82–2.29) | 0.92 (0.46–1.82) |
| **Sex** | | | |
| Men | 1.01 (0.60–1.41) | 1.15 (0.72–1.57) | 0.88 (0.51–1.52) |
| Women | 0.91 (0.39–1.44) | 1.21 (0.62–1.81) | 0.75 (0.36–1.60) |
| **Charlson comorbidity index** | | | |
| < 3 | 0.70 (0.37–1.04) | 0.89 (0.52–1.27) | 0.79 (0.42–1.49) |
| ≥ 3 | 1.77 (0.88–2.66) | 2.10 (1.14–3.06) | 0.84 (0.43–1.66) |
| **Obesity** | | | |
| No | 0.88 (0.49–1.28) | 1.07 (0.64–1.50) | 0.83 (0.45–1.51) |
| Yes[b] | 1.08 (0.55–1.61) | 1.37 (0.78–1.95) | 0.79 (0.41–1.52) |

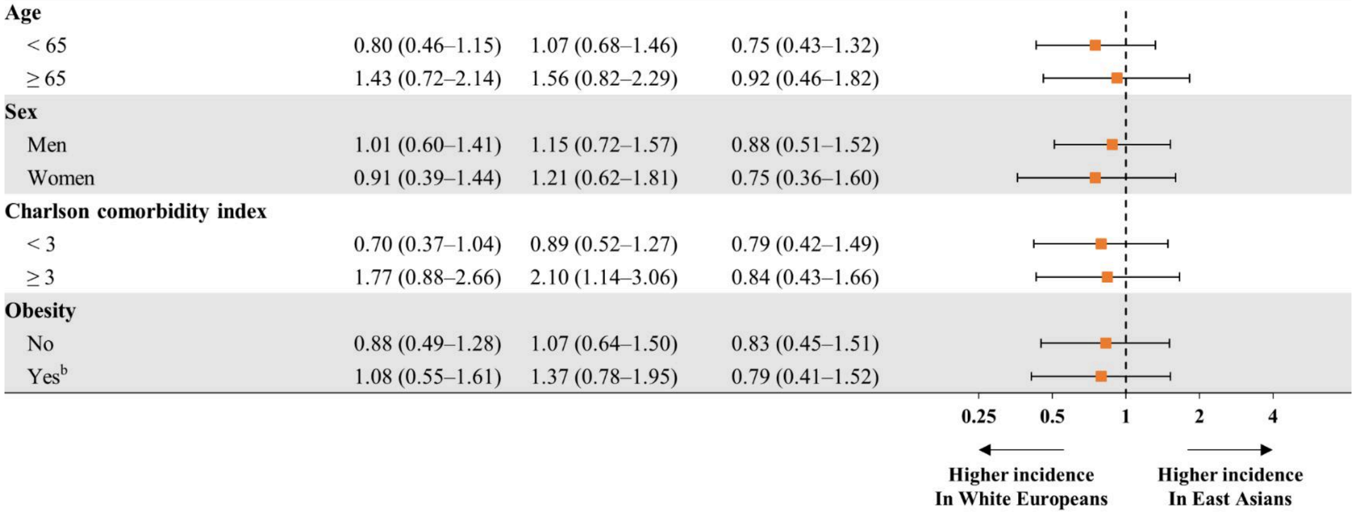


**Figure S5.** Subgroup analyses for major bleeding.

This figure presents the results of subgroup analyses, dividing East Asians and White Europeans into various categories: those under 65 years of age (1411 and 3265, respectively) and those 65 and older (517 and 2652, respectively); men (1197 and 4223, respectively) and women (731 and 1694, respectively); those with a Charlson comorbidity index below 3 (918 and 4903, respectively) and those with an index of 3 or above (662 and 1158, respectively); and participants classified as non-obese (1085 and 3706, respectively) or obese (843 and 2163, respectively). Participants with missing values for the variables under analysis were excluded from the corresponding analysis. Error bars indicate 95% confidence intervals for the incidence rate ratio.

[a]Weighted incidence rates were calculated by dividing the number of first incident events by the total person-years over five years from enrollment, with adjustments for assigned weights. The 95% confidence intervals were estimated using a Poisson distribution and reported per 100 person-years.

[b]Obesity was defined as a body mass index of 25 kg/m² or higher for East Asians, and 30 kg/m² or higher for White Europeans.

CI, condfidence interval; IR, incidence rate; IRR, incidence rate ratio.



Thrombosis and Haemostasis | DOI 10.1055/a-2690-1674 | © The Author(s).

| Intracranial hemorrhage | East Asians Weighted IR[a] (95% CI) | White Europeans Weighted IR[a] (95% CI) | IRR (95% CI) |
|---|---|---|---|
| **Age** | | | |
| < 65 | 0.19 (0.03–0.36) | 0.10 (0.01–0.22) | 1.96 (0.45–8.58) |
| ≥ 65 | 0.70 (0.21–1.20) | 0.26 (0.01–0.56) | 2.69 (0.71–10.20) |
| **Sex** | | | |
| Men | 0.30 (0.08–0.52) | 0.15 (0.01–0.30) | 1.99 (0.57–6.94) |
| Women | 0.41 (0.06–0.76) | 0.12 (0.01–0.31) | 3.29 (0.58–18.54) |
| **Charlson comorbidity index** | | | |
| < 3 | 0.25 (0.05–0.45) | 0.09 (0.01–0.21) | 2.70 (0.59–12.41) |
| ≥ 3 | 0.61 (0.09–1.13) | 0.26 (0.01–0.59) | 2.36 (0.51–10.91) |
| **Obesity** | | | |
| No | 0.28 (0.06–0.50) | 0.18 (0.01–0.35) | 1.61 (0.46–5.68) |
| Yes[b] | 0.41 (0.08–0.73) | 0.13 (0.01–0.30) | 3.21 (0.65–15.94) |

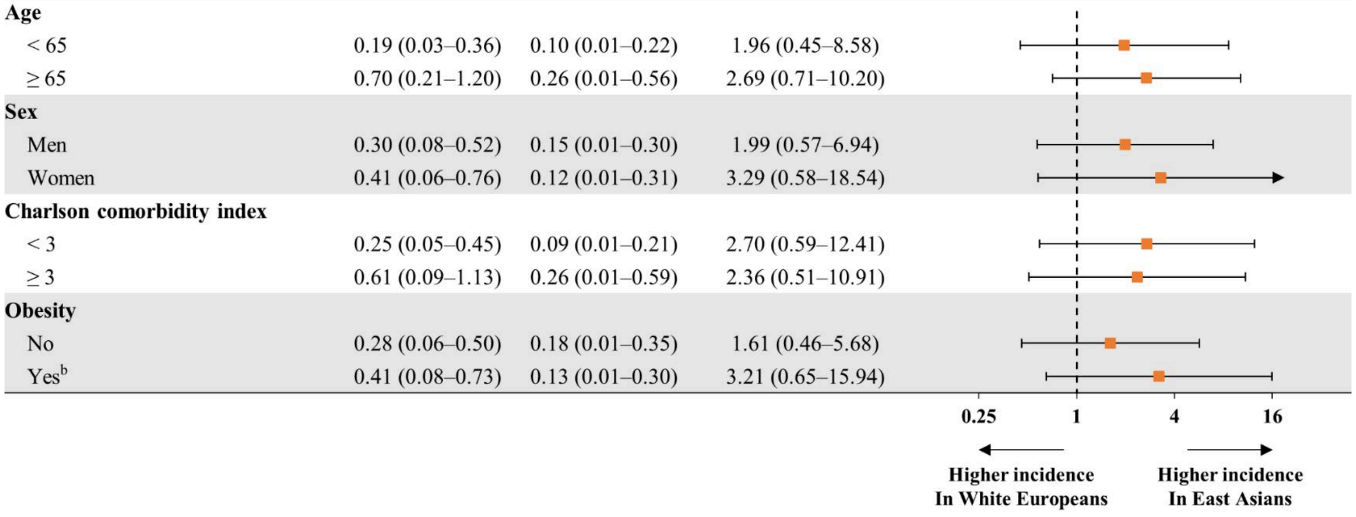


**Figure S6.** Subgroup analyses for intracranial hemorrhage.

This figure presents the results of subgroup analyses, dividing East Asians and White Europeans into various categories: those under 65 years of age (1411 and 3265, respectively) and those 65 and older (517 and 2652, respectively); men (1197 and 4223, respectively) and women (731 and 1694, respectively); those with a Charlson comorbidity index below 3 (918 and 4903, respectively) and those with an index of 3 or above (662 and 1158, respectively); and participants classified as non-obese (1085 and 3706, respectively) or obese (843 and 2163, respectively). Participants with missing values for the variables under analysis were excluded from the corresponding analysis. Error bars indicate 95% confidence intervals for the incidence rate ratio.

[a]Weighted incidence rates were calculated by dividing the number of first incident events by the total person-years over five years from enrollment, with adjustments for assigned weights. The 95% confidence intervals were estimated using a Poisson distribution and reported per 100 person-years.

[b]Obesity was defined as a body mass index of 25 kg/m² or higher for East Asians, and 30 kg/m² or higher for White Europeans.

CI, condfidence interval; IR, incidence rate; IRR, incidence rate ratio.



Thrombosis and Haemostasis | DOI 10.1055/a-2690-1674 | © The Author(s).

| Bleeding from gastrointestinal system | East Asians Weighted IR[a] (95% CI) | White Europeans Weighted IR[a] (95% CI) | IRR (95% CI) |
|---|---|---|---|
| **Age** | | | |
| < 65 | 0.49 (0.22–0.76) | 0.97 (0.59–1.34) | 0.51 (0.26–0.99) |
| ≥ 65 | 0.48 (0.07–0.89) | 1.30 (0.63–1.97) | 0.37 (0.14–0.99) |
| **Sex** | | | |
| Men | 0.58 (0.28–0.89) | 0.99 (0.60–1.39) | 0.58 (0.30–1.13) |
| Women | 0.32 (0.01–0.62) | 1.09 (0.53–1.65) | 0.29 (0.10–0.87) |
| **Charlson comorbidity index** | | | |
| < 3 | 0.39 (0.14–0.65) | 0.80 (0.45–1.16) | 0.49 (0.23–1.07) |
| ≥ 3 | 0.81 (0.21–1.42) | 1.83 (0.93–2.72) | 0.45 (0.18–1.08) |
| **Obesity** | | | |
| No | 0.42 (0.15–0.69) | 0.90 (0.50–1.29) | 0.47 (0.21–1.02) |
| Yes[b] | 0.57 (0.19–0.96) | 1.24 (0.68–1.80) | 0.46 (0.21–1.04) |

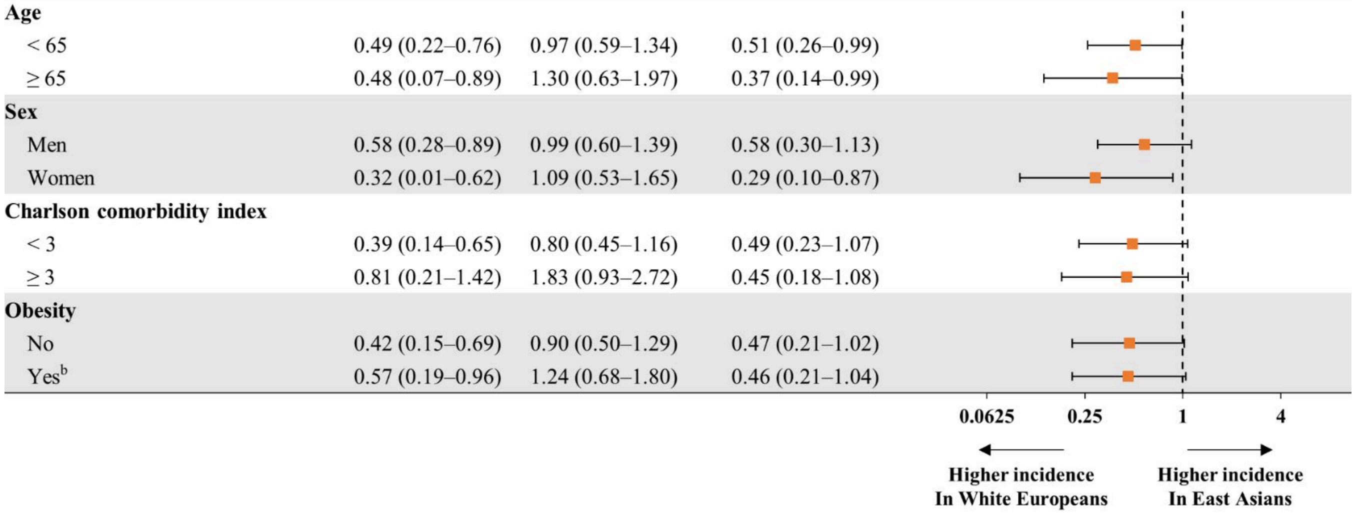


**Figure S7.** Subgroup analyses for bleeding from gastrointestinal system.

This figure presents the results of subgroup analyses, dividing East Asians and White Europeans into various categories: those under 65 years of age (1411 and 3265, respectively) and those 65 and older (517 and 2652, respectively); men (1197 and 4223, respectively) and women (731 and 1694, respectively); those with a Charlson comorbidity index below 3 (918 and 4903, respectively) and those with an index of 3 or above (662 and 1158, respectively); and participants classified as non-obese (1085 and 3706, respectively) or obese (843 and 2163, respectively). Participants with missing values for the variables under analysis were excluded from the corresponding analysis. Error bars indicate 95% confidence intervals for the incidence rate ratio.

[a]Weighted incidence rates were calculated by dividing the number of first incident events by the total person-years over five years from enrollment, with adjustments for assigned weights. The 95% confidence intervals were estimated using a Poisson distribution and reported per 100 person-years.

[b]Obesity was defined as a body mass index of 25 kg/m² or higher for East Asians, and 30 kg/m² or higher for White Europeans.

CI, condfidence interval; IR, incidence rate; IRR, incidence rate ratio.



Thrombosis and Haemostasis | DOI 10.1055/a-2690-1674 | © The Author(s).

| Bleeding from respiratory system | East Asians Weighted IR[a] (95% CI) | White Europeans Weighted IR[a] (95% CI) | IRR (95% CI) |
|---|---|---|---|
| **Age** | | | |
| < 65 | 0.17 (0.01–0.33) | 0.44 (0.19–0.69) | 0.40 (0.13–1.17) |
| ≥ 65 | 0.31 (0.01–0.64) | 0.66 (0.19–1.13) | 0.47 (0.13–1.69) |
| **Sex** | | | |
| Men | 0.28 (0.07–0.49) | 0.58 (0.28–0.88) | 0.48 (0.19–1.20) |
| Women | 0.08 (0.01–0.24) | 0.29 (0.01–0.58) | 0.28 (0.03–2.39) |
| **Charlson comorbidity index** | | | |
| < 3 | 0.18 (0.09–0.34) | 0.26 (0.06–0.47) | 0.67 (0.20–2.27) |
| ≥ 3 | 0.28 (0.01–0.63) | 0.90 (0.28–1.53) | 0.31 (0.07–1.29) |
| **Obesity** | | | |
| No | 0.18 (0.01–0.36) | 0.54 (0.23–0.84) | 0.35 (0.12–1.06) |
| Yes[b] | 0.26 (0.01–0.52) | 0.50 (0.15–0.85) | 0.53 (0.16–1.76) |

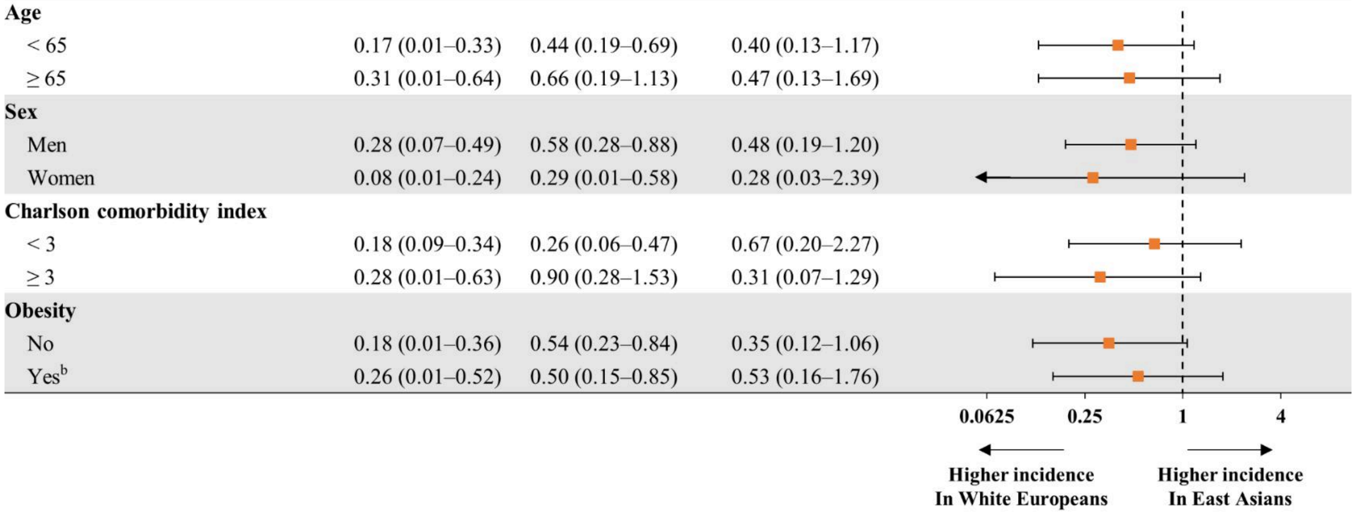


**Figure S8.** Subgroup analyses for bleeding from respiratory system.

This figure presents the results of subgroup analyses, dividing East Asians and White Europeans into various categories: those under 65 years of age (1411 and 3265, respectively) and those 65 and older (517 and 2652, respectively); men (1197 and 4223, respectively) and women (731 and 1694, respectively); those with a Charlson comorbidity index below 3 (918 and 4903, respectively) and those with an index of 3 or above (662 and 1158, respectively); and participants classified as non-obese (1085 and 3706, respectively) or obese (843 and 2163, respectively). Participants with missing values for the variables under analysis were excluded from the corresponding analysis. Error bars indicate 95% confidence intervals for the incidence rate ratio.

[a]Weighted incidence rates were calculated by dividing the number of first incident events by the total person-years over five years from enrollment, with adjustments for assigned weights. The 95% confidence intervals were estimated using a Poisson distribution and reported per 100 person-years.

[b]Obesity was defined as a body mass index of 25 kg/m² or higher for East Asians, and 30 kg/m² or higher for White Europeans.

CI, condfidence interval; IR, incidence rate; IRR, incidence rate ratio.



Thrombosis and Haemostasis | DOI 10.1055/a-2690-1674 | © The Author(s).

| Bleeding from genitourinary system | East Asians Weighted IR[a] (95% CI) | White Europeans Weighted IR[a] (95% CI) | IRR (95% CI) |
|---|---|---|---|
| **Age** | | | |
| < 65 | 0.27 (0.07–0.47) | 0.69 (0.38–1.01) | 0.40 (0.17–0.94) |
| ≥ 65 | 0.35 (0.01–0.70) | 0.90 (0.35–1.45) | 0.39 (0.12–1.26) |
| **Sex** | | | |
| Men | 0.30 (0.08–0.52) | 0.81 (0.46–1.17) | 0.37 (0.16–0.87) |
| Women | 0.32 (0.01–0.63) | 0.64 (0.21–1.07) | 0.50 (0.15–1.60) |
| **Charlson comorbidity index** | | | |
| < 3 | 0.11 (0.01–0.24) | 0.68 (0.35–1.01) | 0.16 (0.04–0.59) |
| ≥ 3 | 0.37 (0.01–0.78) | 0.94 (0.31–1.57) | 0.40 (0.11–1.42) |
| **Obesity** | | | |
| No | 0.21 (0.02–0.41) | 0.76 (0.40–1.12) | 0.28 (0.10–0.78) |
| Yes[b] | 0.43 (0.10–0.76) | 0.73 (0.30–1.15) | 0.59 (0.23–1.56) |

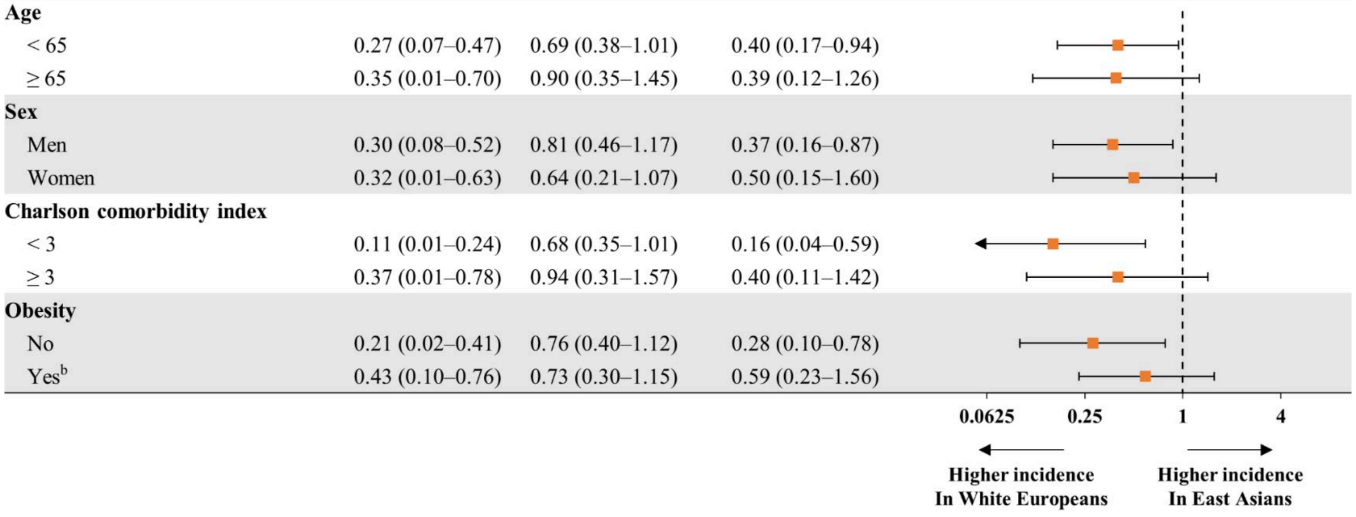


**Figure S9.** Subgroup analyses for bleeding from genitourinary system.

This figure presents the results of subgroup analyses, dividing East Asians and White Europeans into various categories: those under 65 years of age (1411 and 3265, respectively) and those 65 and older (517 and 2652, respectively); men (1197 and 4223, respectively) and women (731 and 1694, respectively); those with a Charlson comorbidity index below 3 (918 and 4903, respectively) and those with an index of 3 or above (662 and 1158, respectively); and participants classified as non-obese (1085 and 3706, respectively) or obese (843 and 2163, respectively). Participants with missing values for the variables under analysis were excluded from the corresponding analysis. Error bars indicate 95% confidence intervals for the incidence rate ratio.

[a]Weighted incidence rates were calculated by dividing the number of first incident events by the total person-years over five years from enrollment, with adjustments for assigned weights. The 95% confidence intervals were estimated using a Poisson distribution and reported per 100 person-years.

[b]Obesity was defined as a body mass index of 25 kg/m² or higher for East Asians, and 30 kg/m² or higher for White Europeans.

CI, condfidence interval; IR, incidence rate; IRR, incidence rate ratio.



Thrombosis and Haemostasis | DOI 10.1055/a-2690-1674 | © The Author(s).
